# The IL-33/ST2 pathway shapes the regulatory T cell phenotype to promote intestinal cancer

**DOI:** 10.1038/s41385-019-0176-y

**Published:** 2019-06-05

**Authors:** Eva Pastille, Marie-Hélène Wasmer, Alexandra Adamczyk, Vivian P. Vu, Lukas F. Mager, Nhi Ngo Thi Phuong, Vittoria Palmieri, Cedric Simillion, Wiebke Hansen, Stefan Kasper, Martin Schuler, Beat Muggli, Kathy D. McCoy, Jan Buer, Inti Zlobec, Astrid M. Westendorf, Philippe Krebs

**Affiliations:** 1 Institute of Medical Microbiology, University Hospital Essen, University of Duisburg-Essen, Essen, Germany; 2 Institute of Pathology, University of Bern, Bern, Switzerland; 3 Graduate School for Cellular and Biomedical Sciences, University of Bern, Bern, Switzerland; 4 Department of Physiology and Pharmacology, Snyder Institute of Chronic Diseases, Cumming School of Medicine, University of Calgary, Calgary, Canada; 5 Department of Clinical Research, University of Bern, Bern, Switzerland; 6 Interfaculty Bioinformatics Unit and Swiss Institute of Bioinformatics, University of Bern, Bern, Switzerland; 7 Department of Medical Oncology, West German Cancer Center, University Hospital Essen, University Duisburg-Essen, Essen, Germany; 8 German Cancer Consortium (DKTK), Partner site University Hospital Essen, Essen, Germany; 9 Department of Visceral Surgery and Medicine, Inselspital, Bern University Hospital, University of Bern, Bern, Switzerland

## Abstract

The composition of immune infiltrates strongly affects the prognosis of patients with colorectal cancer (CRC). Interleukin (IL)-33 and regulatory T cells (Tregs) in the tumor microenvironment have been separately implicated in CRC; however their contribution to intestinal carcinogenesis is still controversial. Here, we reveal that IL-33 signaling promotes CRC by changing the phenotype of Tregs. In mice with CRC, tumor-infiltrating Tregs preferentially upregulate IL-33 receptor (ST2), and IL-33/ST2 signaling positively correlates with tumor number and size. Transcriptomic and flow cytometry analyses demonstrate that ST2 expression induces a more activated and migratory phenotype in FOXP3^+^ Tregs, which favors their accumulation in the tumor environment. Consequently, genetic ablation of *St2* reduces Treg infiltration and concomitantly enhances the frequencies of effector CD8^+^ T cells, thereby restraining CRC. Mechanistically, IL-33 curtails IL-17 production by FOXP3^+^ Tregs and inhibits Th17 differentiation. In humans, numbers of activated ST2-expressing Tregs are increased in blood and tumor lesions of CRC patients, suggesting a similar mode of regulation. Together, these data indicate a central role of IL-33/ST2 signaling in shaping an immunosuppressive environment during intestinal tumorigenesis. Blockade of this pathway may provide a strategy to modulate the composition of CRC immune infiltrates.

## Introduction

Interleukin-33 (IL-33) is an IL-1 family member, which mediates its biological effects via binding to a heterodimeric receptor complex formed by IL-1RL1 (or ST2) and its co receptor, IL-1 receptor accessory protein (IL-1RAP).^1^ IL-33 is constitutively expressed as a nuclear factor by a broad range of cell types, including fibroblasts, epithelial, and endothelial cells, particularly in mucosal tissues.^2^ However, upon tissue injury, cell stress or necrosis IL-33 can be released to act as an alarmin by activating cells of lymphoid and myeloid origin.^3^

The IL-33/ST2 pathway was originally described to play a key role in type 2 immunity via activation of ST2-expressing T helper 2 (Th2) cells.^4^ Besides this Th2-promoting function of IL-33, recent murine studies provided evidence for a pivotal role of the IL-33/ST2 pathway on the biology of regulatory T cells (Tregs). More precisely, in vivo administration of recombinant IL-33 induces a ST2-dependent proliferation and accumulation of FOXP3^+^ Tregs in the thymus and in the periphery. Mechanistically, IL-33 directly acts on ST2^+^ FOXP3^+^ Tregs but also stimulates IL-2 production by CD11c^+^ dendritic cells, which in turn facilitate the expansion of FOXP3^+^ Tregs.^5,6^ Furthermore, IL 33 signaling in FOXP3^+^ Tregs is important for their stability and suppressive function in vivo.^5^

Tregs are critical for the maintenance of immune homeostasis and the prevention of autoimmunity by exerting various immunosuppressive mechanisms towards self-reactive T cells.^7^ Conversely, in solid tumors Tregs counteract antitumor immunity. This explains why high amounts of peripheral or tumor-infiltrating Tregs are often associated with poor prognosis in various cancer entities.^8^ However, the role of Tregs for colorectal cancer (CRC) is ambiguous.

In the intestine, dietary antigens or antigens of commensal gut bacteria constantly trigger inflammatory processes, which can increase the risk of cancer development if they are not tightly regulated. By controlling this physiological intestinal inflammation, Tregs maintain immune tolerance, thereby reducing the risk of inflammation-associated tumorigenesis.^9^ Accordingly, several studies identified a positive correlation between high frequencies of tumor-infiltrating FOXP3^+^ Treg and an improved survival of CRC patients.^10^ Nevertheless, FOXP3^+^ Tregs exert potent suppressive functions towards effector T cells, thereby creating an immunosuppressive environment which may influence the clinical outcome of CRC patients.^11^ Such opposing contribution of Tregs to CRC may rely on the ability of Tregs to undergo functional plasticity.^12^ Indeed, Tregs are for instance able to adapt to the tissue environment and acquire a Th1− or Th17− effector phenotype while retaining their suppressive function (reviewed in ref. ^13^). While the cytokine signals driving T helper cell differentiation into different subsets are well-characterized, the nature of the inflammatory cues released by the environment to govern Treg plasticity and function are still elusive.^14^

During intestinal tumorigenesis, IL-33 protein expression is induced in transformed epithelial cells.^15,16^ We previously reported that IL-33 signaling stimulates the production of pro-tumorigenic IL-6, and that ST2-deficient mice show a delayed tumor growth in the colon.^16^ Several studies support these findings of a tumor-promoting role of IL-33/ST2 signaling for intestinal tumorigenesis, also using different animal models.^15,17^ However, the identity of the ST2-expressing cells in CRC lesions and their function during intestinal tumorigenesis is yet to be clearly defined.

In this study, we investigated the impact of IL-33/ST2 signaling on immune cell function in CRC. Our findings using mouse studies and patient-derived samples indicate that IL-33 critically regulates the functional phenotype and the number of Treg in the CRC environment. This in turn restricts effector CD8^+^ T cell immunity and promotes tumorigenesis in the colon.

## Results

### Upregulation of *Il33* and *St2* expression in murine CRC

**Fig. 1:**
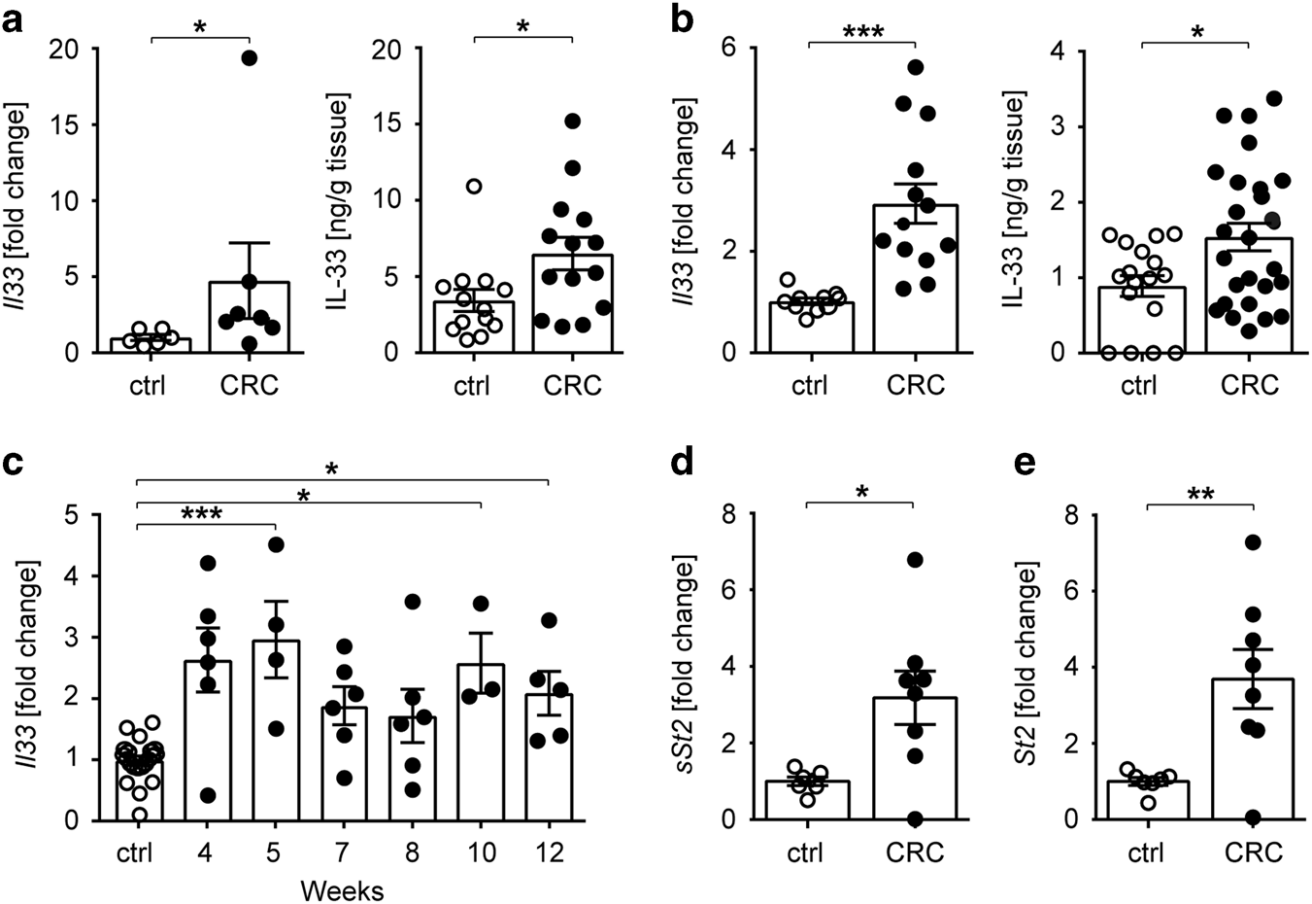
The IL-33/ST2 pathway is upregulated in intestinal tumors. Mice were treated with AOM/DSS and cancerous colonic tissues (CRC) were isolated for analysis after 10–12 weeks or at the indicated time points. Unless otherwise indicated, colon tissues from naïve mice of the corresponding strain were used as control (ctrl). *Il33* transcript levels (left panel) and IL-33 protein secretion from explant cultures (right panel) were measured in the colon of **a** C57BL/6 mice (*n* = 6–17 mice per group; data pooled from two, respectively, four independent experiments) or **b** BALB/c mice (*n* = 10–26 mice per group; data pooled from three, respectively, five independent experiments). **c** *Il-33* transcript levels were measured in tumor tissues of BALB/c mice at the indicated time points after the first AOM injection. Ctrl, *n* = 21; CRC, *n* = 3-6 mice per group; data are from two experiments. Transcript levels of **d** *sSt2* and **e** *St2* were measured in tumor lesions (CRC, *n* = 8 mice per group) or adjacent tumor-free tissues (ctrl, *n* = 7 mice per group) from C57BL/6 mice. Data are from one experiment. Data are mean ± SEM. Statistical analyses were performed using **a**, **b**, **d**, **e** standard Student’s *t-*test or **c** one-way ANOVA with Dunnett’s post-test. *P < 0.05; **P < 0.01; ***P < 0.001

IL-33 expression has been shown to become upregulated upon neoplastic transformation of human and murine colonic tissues and to promote intestinal tumorigenesis.^15,16^ To address the role of the IL-33/ST2 axis on immune cells in CRC, we treated C57BL/6 and BALB/c mice with azoxymethane (AOM)/dextran sulfate sodium (DSS). Combined application of these chemicals induces dysplastic changes in the mouse colon that feature many of the histological and molecular characteristics of human adenocarcinomas.^18^ Independent of the genetic background, we found that *Il33* transcripts and IL-33 protein were increased in malignant colonic tissues (Fig. 1a, b). Interestingly, colonic *Il33* transcripts were increased as early as 5 weeks after treatment start, with levels remaining 2.5-fold higher than untreated control mice until the end of the treatment (Fig. 1c). In addition, expression of both the soluble (*sSt2*) and transmembrane forms of *St2* (*St2*) were upregulated in tumor lesions versus adjacent tumor-free colons of C57BL/6 mice (Fig. 1d, e).

### Tumor-infiltrating CD4^+^ FOXP3^+^ Tregs preferentially express ST2

**Fig. 2:**
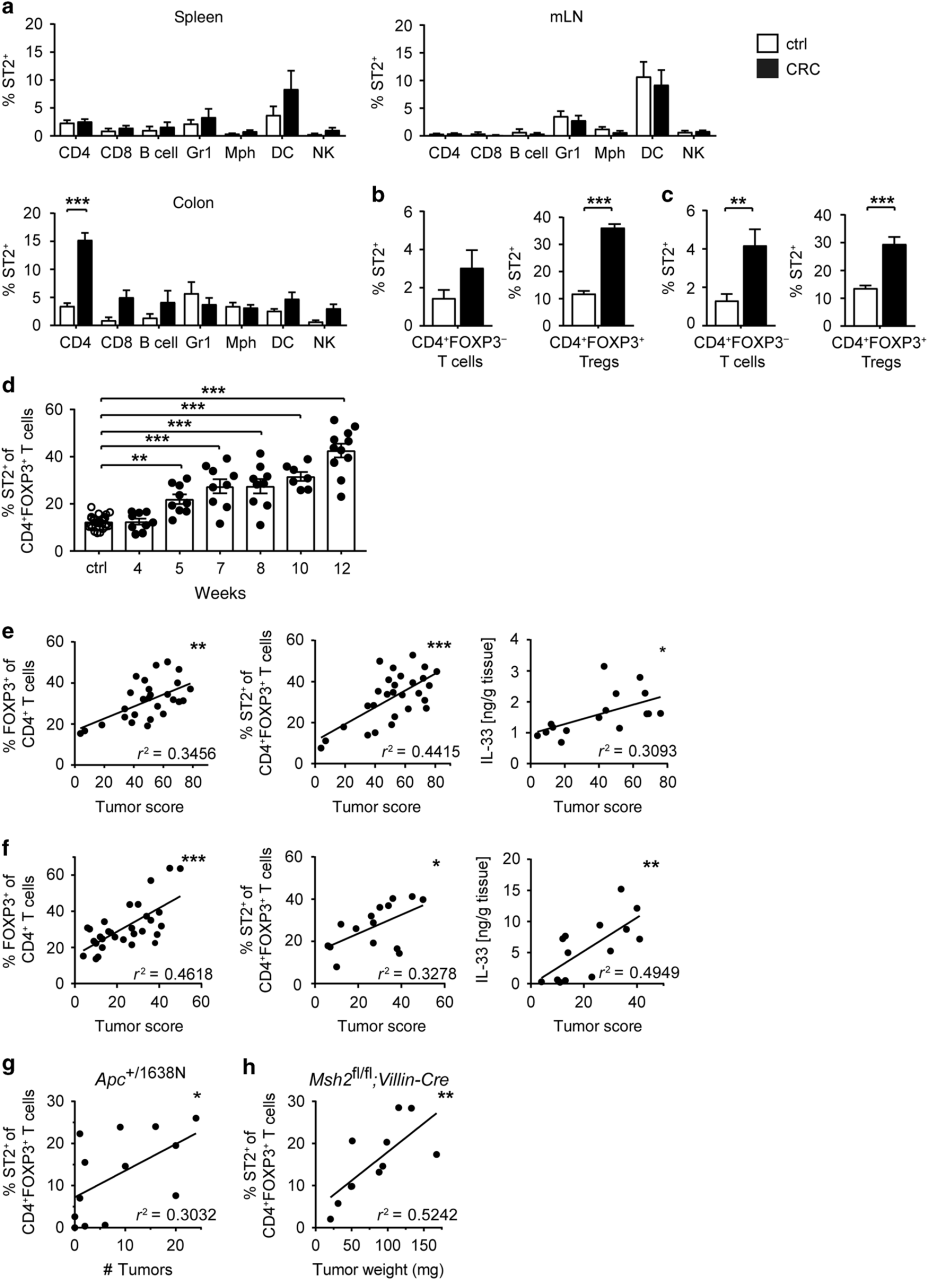
ST2 is preferentially upregulated on CD4^+^ FOXP3^+^ Tregs in intestinal tumors. Mice were treated with AOM/DSS (CRC; black bars) or left untreated (ctrl; white bars), and immune cells from spleen, mesenteric lymph nodes (mLN), or colon were analyzed by flow cytometry. **a** Frequencies of ST2-positive cells were measured among CD4^+^ T cells, CD8^+^ T cells, B220^+^ B cells, GR1^+^ granulocytes, F4/80^+^ CD11c^int/−^ macrophages (Mph), CD11c^+^F4/80^−^ dendritic cells (DC) and CD335^+^ NK cells, respectively (*n* = 7–8 BALB/c mice per group). Frequencies of ST2-positive cells were measured among FOXP3^−^ (left panel) or FOXP3^+^ (right panel) CD4^+^ T cells from **b** BALB/c (*n* = 15–22 mice per group) or **c** C57BL/6-*Foxp3*/RFP mice (*n* = 10–16 mice per group). **d** Frequencies of ST2^+^ FOXP3^+^ Tregs were measured in CRC lesions of BALB/c mice at the indicated time points during AOM/DSS treatment (*n* = 26 for ctrl and *n* = 7–11 for CRC mice per time point). FOXP3^+^ Treg frequencies (left panel), ST2-expressing FOXP3^+^ Treg frequencies (middle panel), or IL-33 protein expression (right panel) in colon were correlated with tumor score in **e** BALB/c (*n* = 16-28) or **f** C57BL/6-*Foxp3*/RFP (*n* = 14–31) mice. **g** Alternatively, *Apc*^+/1638N^ mice (*n* = 13) were analyzed at different ages and frequencies of ST2-expressing FOXP3^+^ Treg were correlated with tumor numbers in the colon. **h** *Msh2*^fl/fl^;*Villin-Cre* mice (*n* = 11) were analyzed 269–342 days after birth and frequencies of ST2-expressing FOXP3^+^ Treg were correlated with tumor weight in the small intestine. Data are mean ± SEM and were pooled from **a**, **h** two, **b**, **c** four, **d** three, **e** 4–5, **f** 3–7 or **g** several independent experiments. Statistical analyses were performed using **a–c** two-way ANOVA with Sidak post-test and **d** one-way ANOVA with Dunnett's post-test. Correlations were calculated using Spearman correlation analysis. *P < 0.05; **P < 0.01; ***P < 0.001

IL-33 signals via its unique receptor ST2, which is expressed on several cell types.^3^ To identify the cells potentially reacting to IL-33 in the tumor microenvironment, we next measured ST2 expression on tumor-infiltrating immune cells. Compared to untreated controls, frequencies of ST2-expressing cells in the colon of CRC mice were higher among T cells and NK cells, yet not among B cells, granulocytes, macrophages, or dendritic cells. Moreover, frequencies of ST2-expressing cells remained unchanged among splenocytes or mesenteric lymph node (mLN) cells of AOM/DSS-treated mice (Fig. 2a). Tumor-infiltrating CD4^+^ T cells showed the highest relative ratio of ST2-expressing cells and a substantial proportion of CD4^+^ T cells in CRC lesions were FOXP3^+^ Tregs (Supplementary Fig. 1A and B). Hence, we further characterized ST2 expression among CD4^+^ T cell subsets. Notably, more than 30% of CD4^+^ FOXP3^+^ Tregs in colonic tumors of BALB/c mice expressed ST2, versus 3% of CD4^+^ FOXP3^-^ T cells (Fig. 2b), thus indicating that ST2 expression in the CD4^+^ T cell compartment was mainly restricted to FOXP3^+^ Tregs. These findings were also validated in C57BL/6 intestinal tumors (Fig. 2c).

We previously demonstrated that CD4^+^ FOXP3^+^ Tregs promote tumor progression in the AOM/DSS-induced model of CRC.^19^ To address the specific role of ST2-expressing Tregs in intestinal tumorigenesis, we next performed a kinetic analysis of colonic ST2^+^ CD4^+^ FOXP3^+^ Tregs, which were found to progressively increase in frequency and transiently upregulate the IL-33 receptor during the course of AOM/DSS treatment (Fig. 2d and Supplementary Fig. 1C, respectively). In addition, the frequency of CD4^+^ FOXP3^+^ Tregs, the proportion of ST2^+^ CD4^+^ FOXP3^+^ Tregs, and IL-33 protein expression in the colon all positively correlated with tumor scores (i.e. the sum of the relative size of all tumors in a given mouse), irrespective of the genetic background (Fig. 2e, f). Importantly, frequency of ST2^+^ CD4^+^ FOXP3^+^ Tregs—but not of CD4^+^ FOXP3^+^ Tregs—also correlated with intestinal tumorigenesis in other murine models of CRC based on mutation in the *Apc* gene or on intestinal-specific deficiency in mismatch repair (Fig. 2g, h and Supplementary Fig. 1D–G). Together, these data clearly pointed toward a contribution of IL-33/ST2 signaling to CRC. Moreover, the gradual upregulation of ST2 on tumor-isolated CD4^+^ FOXP3^+^ Tregs during intestinal tumorigenesis suggested an immune regulatory function of IL-33 for this disease.

### ST2 deficiency leads to reduced Treg frequencies in colonic tumor tissues

**Fig. 3:**
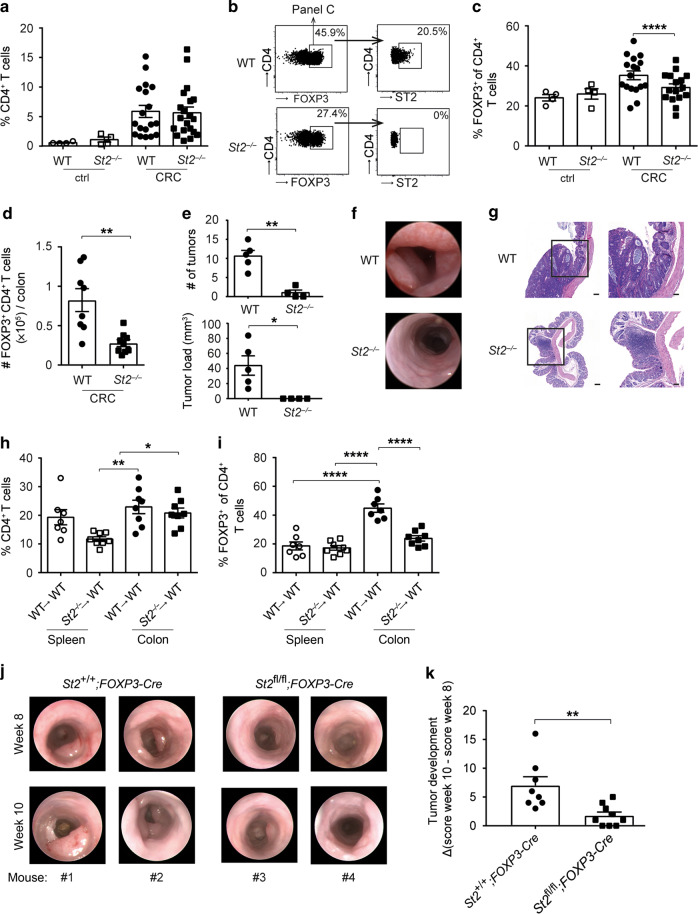
Treg frequencies are reduced in tumors of *St2*^−/−^ mice. Wild-type (WT) or *St2*^*−/−*^ C57BL/6 mice were treated with AOM/DSS (CRC) or left untreated (ctrl) and **a** frequencies of CD4^+^ T cells in colonic tissues were measured after 10 weeks (*n* = 4–20 mice per group). **b** Flow cytometry plots from one representative CRC mouse per group depicted in **c** showing frequencies of CD4^+^ FOXP3^+^ Tregs (panels on the left) and frequencies of ST2-expressing cells among FOXP3^+^ Tregs (panels on the right). **c** Frequencies of FOXP3^+^ Treg were measured among CD4^+^ T cells based on the gating strategy shown in **b**. **d** Absolute numbers of FOXP3^+^ Treg were measured in the colon of AOM/DSS-treated mice (*n* = 8–9 mice per group). **e** Tumor numbers and tumor load were assessed in the colon of CRC mice (*n* = 5 mice per group). **f** Endoscopy pictures showing tumors in the distal colon from one representative CRC mouse per group depicted in **e**. **g** Hematoxylin and eosin (H&E) sections displaying representative colon tumors in the indicated groups of mice. Scale bars: overview: 200 μm; inlay: 100 μm. Indicated sets of chimeric mice were treated with AOM/DSS and **h** frequencies of CD4^+^ T cells or **i** frequencies of CD4^+^ FOXP3^+^ Treg were measured in the indicated organs after 10–13 weeks (*n* = 7–8 mice per group). **j** Intestinal tumorigenesis was assessed by endoscopy and **k** tumor development was calculated in the indicated groups of mice by subtracting tumor score on week 10 from tumor score on week 8 after the start of AOM/DSS treatment (*n* = 8–9 mice per group). Data are mean ± SEM and show **a**, **c** four pooled independent experiments out of six or **e** one representative experiment out of three. **d**, **h**, **i**, **k** data were pooled from two independent experiments. Statistical analyses were performed using **a**, **c–e**, **k** standard Student’s *t-*test or **h**, **i** one-way ANOVA with Bonferroni post-test. *P < 0.05; **P < 0.01; ****P < 0.0001

To evaluate the role of IL-33/ST2 signaling on CD4^+^ FOXP3^+^ Tregs for intestinal tumorigenesis, we treated C57BL/6 wild-type (WT) and *St2*^*−/−*^ mice with AOM and DSS. Compared to naïve controls, frequencies of CD4^+^ T cells were elevated in the CRC tissue, yet similar between the two groups (Fig. 3a). Importantly, proportions and absolute numbers of CD4^+^ FOXP3^+^ Tregs were lower in *St2*^*−/−*^ versus WT CRC lesions, although ST2 deficiency per se had no impact on Treg ratios in the intestine of naïve mice (Fig. 3b–d). In line with these results, there were fewer and smaller intestinal tumors in *St2*^*−/−*^ mice, which was further corroborated by endoscopic analysis and histology (Fig. 3e–g).

Next, we created bone marrow (BM) chimeric mice to assess the contribution of *St2* in the radio-sensitive/hematopoietic compartment to the accumulation of Tregs in CRC. AOM/DSS-treated chimeras with ST2-deficient hematopoietic cells had no alteration in CD4^+^ T cell frequencies, yet they showed a reduction of CD4^+^ FOXP3^+^ Tregs in intestinal tumors (Fig. 3h, i).

To also address the specific contribution of ST2 on CD4^+^ FOXP3^+^ Tregs to CRC, we generated mixed BM chimeras by reconstituting WT recipient mice with equal ratios of BM cells from WT and *Foxp3*^*DTR*^ mice (WT:*Foxp3*^*DTR*^) or from *St2*^*−/−*^ and *Foxp3*^*DTR*^ animals (*St2*^*−/−*^:*Foxp3*^*DTR*^). Diphtheria toxin-induced FOXP3^+^ cell depletion during colon tumorigenesis in these chimeras provided a model in which Tregs present in the reconstituted (*St2*^*−/−*^: *Foxp3*^*DTR*^) animals were the only cell subset being entirely deficient for ST2, while control (WT: *Foxp3*^*DTR*^) mice harbored ST2-proficient Tregs (Supplementary Fig. 2A). Longitudinal endoscopic studies performed before and after the period of diphtheria toxin application in these chimeras revealed that presence of ST2-competent Tregs during CRC development promotes enhanced tumor formation compared to ST2-deficient Tregs (Supplementary Fig. 2B). To strengthen these results, *St2*^*fl/fl*^*;Foxp3-Cre* mice, in which ST2 is specifically deleted in Tregs, were next subjected to AOM/DSS treatment. In line with the data from the BM chimera experiments, endoscopic analysis after 8 and 10 weeks revealed fewer and smaller intestinal tumors in *St2*^*fl/fl*^*;Foxp3-Cre* versus *St2*^*+/+*^*;Foxp3-Cre* mice (Fig. 3j). In addition, mice with Treg-specific *St2* ablation showed delayed tumor progression compared to controls with ST2-competent Tregs (Fig. 3k).

These results imply that, during DSS/AOM-induced CRC, IL-33/ST2 signaling is activated in CD4^+^ FOXP3^+^ Tregs to promote their accumulation in the colon. Moreover, the concomitant reduction in *St2*^*−/−*^ mice of tumor load and CD4^+^ FOXP3^+^ Tregs and the findings that Treg-restricted *St2* ablation is associated with reduced CRC development led us to hypothesize that ST2^+^ CD4^+^ FOXP3^+^ Tregs support CRC development.

### Distinct transcriptional profile of ST2-expressing FOXP3^+^ Tregs in murine CRC

**Fig. 4:**
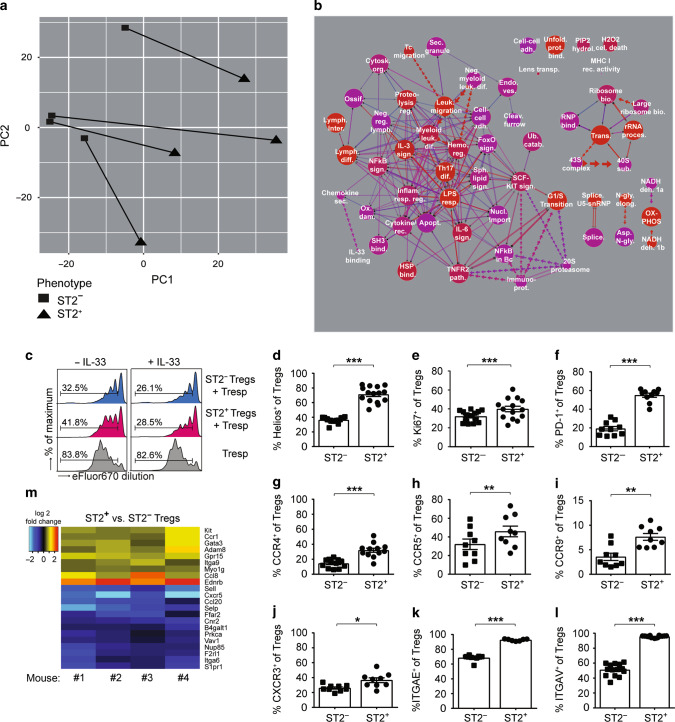
ST2 expression shapes the transcriptome and the phenotype of tumor-derived FOXP3^+^ Tregs. *Foxp3*/eGFP mice were treated with AOM/DSS and eGFP^+^ CD4^+^ T cells from CRC lesions were sort-purified into ST2^+^ versus ST2^−^ cells for microarray analysis. **a** Principal component analysis depicting matched samples, connected by lines, from four analyzed mice. **b** Pathway analysis showing 58 pathways that were found to be differentially regulated between ST2^+^ versus ST2^−^ eGFP^+^ CD4^+^ T cells (with adjusted *p*-value *p* < 0.001 and corrected *p*-value *p* < 0.001). Graph key: red color indicates increased significance and blue (purple) no significance. Size of circles is proportional to the number of genes within a pathway. Connection lines indicate pathways that share genes. Thickness of connecting lines is proportional to the number of genes shared between two pathways. Double lines indicate that the intersection between two pathways is significant. Dotted arrow lines indicate that a particular pathway corresponds to a subset within another pathway. Line length has no particular meaning. Full pathway names, detailed results, and references for the different pathways are shown in Supplementary Table 1. **c** To assess their suppressive capability, sort-purified ST2^−^ and ST2^+^ eGFP^+^ Tregs were co-cultured at a 1:2 ratio with anti-CD3-activated responder *St2*^−*/−*^ T cells (Tresp), together with *St2*^*−/−*^ antigen-presenting cells, in the presence or absence of IL-33. Proliferation of Tresp was analyzed after 3 days, based on the extent of eFluor670 fluorescence dilution. Histograms show one representative out of three independent experiments. **d–l** ST2^−^ and ST2^+^ Tregs (defined as CD4^+^ FOXP3^+^ T cells) were analyzed for expression of the indicated markers (*n* = 7–15 mice per group, pooled from 2 to 3 independent experiments). **m** Heat map showing all genes in the gene ontology pathway “leukocyte migration” (GO:0050900) that are differentially expressed in ST2^+^ versus ST2^−^ eGFP^+^ Tregs isolated from CRC lesions from the indicated four mice (adjusted *p*-value *p* ≤ 0.05). Blue indicates higher transcript expression in ST2^–^ Tregs and red higher expression in ST2^+^ Tregs. **d–l** Data are mean ± SEM and statistical analyses were performed using paired Student’s *t-*test. *P < 0.05; **P < 0.01; ***P < 0.001

As frequencies of ST2^+^ CD4^+^ FOXP3^+^ Tregs in CRC tissue correlated with tumor scores, we next performed a transcriptomic analysis to better understand the role of ST2 for Treg function during intestinal tumorigenesis. Principal component analysis (PCA) of microarray data indicated a clear segregation between ST2^+^ and ST2^−^ CD4^+^ FOXP3^+^ cells isolated from the colon of AOM/DSS-treated *Foxp3*/eGFP reporter mice (Fig. 4a). Moreover, further analysis showed that multiple pathways were differentially regulated in ST2^+^ versus ST2^−^ CD4^+^ FOXP3^+^ T cells, in particular pathways involved in lymphocyte differentiation, regulation, or migration (Fig. 4b and Supplementary Fig. 3A). Taken together, these findings provide evidence that ST2 expression endows CD4^+^ FOXP3^+^ Tregs with distinct phenotypic and possibly functional features.

### ST2 expression modulates the activation but not the suppressive capacity of Tregs

In the following, we studied potential functional consequences of the specific transcription pattern identified in ST2-expressing CD4^+^ FOXP3^+^ Tregs. First, we compared the capacity of isolated tumor-infiltrating ST2^+^ versus ST2^−^ CD4^+^ FOXP3^+^ Tregs to suppress CD4^+^ responder T cells from naive *St2*^*−/−*^ donor mice in vitro. In these settings, ST2 expression did not affect Treg suppressive activity. However, addition of recombinant murine IL-33 (rmIL-33) to the culture enhanced the suppressive capacity of CD4^+^ FOXP3^+^ Tregs (Fig. 4c). Next, we applied flow cytometry analysis for phenotyping. This analysis revealed that, compared to ST2^−^ counterpart, ST2^+^ CD4^+^ FOXP3^+^ Tregs expressed higher levels of IKZF2/Helios, thus indicating a more stable regulatory activity^20^ and a thymic origin^21^ (Fig. 4d). The proliferation marker Ki67 and the inhibitory receptor PD-1 (which is encoded by *Pdcd1*) were also upregulated in ST2^+^ CD4^+^ FOXP3^+^ Tregs (Fig. 4e, f). Moreover, several chemokine receptors including CCR4, CCR5, CCR9, CXCR3, and the integrins α-E/CD103 and α-V (which are encoded by *Itgae* and *Itgav*, respectively) showed increased expression on ST2^+^ versus ST2^−^ CD4^+^ FOXP3^+^ Tregs isolated from CRC lesions (Fig. 4g–l). In agreement with these findings, the ligands of these chemokine receptors and integrins were more abundant in CRC lesions of WT versus *St2*^*−/−*^ mice (Supplementary Fig. 3B–F).

These results supported the notion that ST2^+^ CD4^+^ FOXP3^+^ Tregs are not primarily induced in colonic tumors, but rather migrate and proliferate at the site of IL-33 expression. Indeed, detailed analysis of the ontology pathway “leukocyte migration” indicated several migration-related genes that were differentially expressed in colonic ST2^+^ versus ST2^−^ CD4^+^ FOXP3^+^ Tregs. In particular, *endothelin receptor type B* (*Ednrb*), *Ccr1*, *Gpr15*, *myosin 1G* (*Myo1g*), and *integrin α-9* (*Itga9*) were upregulated in ST2^+^ CD4^+^ FOXP3^+^ Tregs (Fig. 4m). These genes or the ligands of their products have been involved in intestinal inflammation or CRC development,^22–26^ which suggests an active migration of ST2^+^ Tregs to the tumor tissue.

### IL-33 restrains IL-17 production in CD4^+^ T cells

**Fig. 5:**
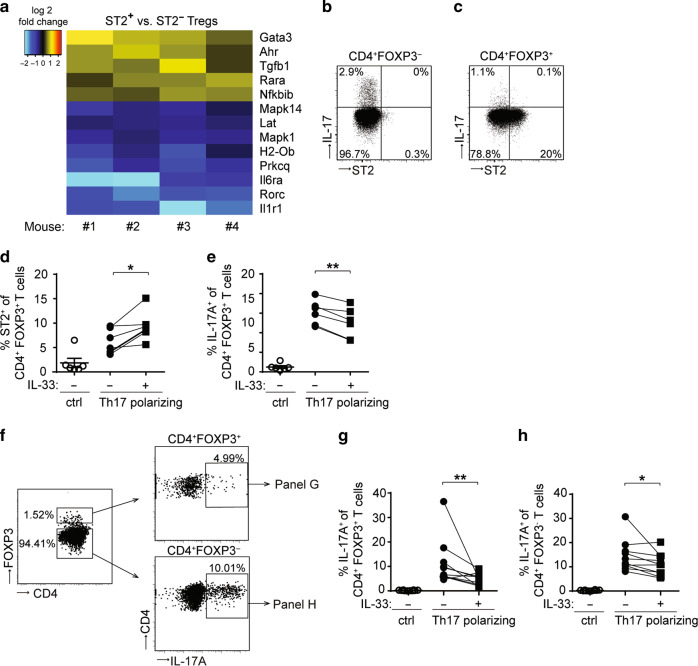
IL-33 restrains IL-17 production in CD4^+^ T cells. **a** *Foxp3*/eGFP mice were treated with AOM/DSS and eGFP^+^ CD4^+^ T cells were isolated from CRC lesions for transcriptomic analysis. Heat map showing all genes in the gene ontology pathway “Th17 cell differentiation” (ko04659) that are differentially expressed in ST2^+^ versus ST2^−^ eGFP^+^ Tregs isolated from CRC lesions from the indicated four mice (adjusted *p*-value *p* ≤ 0.05). Blue indicates higher transcript expression in ST2^−^ Tregs and yellow/red higher expression in ST2^+^ Tregs. BALB/c mice were treated with AOM/DSS and FOXP3^−^ CD4^+^ T cells **b** or FOXP3^+^ CD4^+^ Tregs **c** were analyzed in the intestine for ST2 and IL-17 expression. Shown are flow cytometry plots from each one representative sample out of three. **d–h** Sort-purified eGFP^+^ **b**, **c** or eGFP^−^ **d**, **f** CD4^+^ T cells from spleens of naïve *Foxp3*/eGFP reporter mice were stimulated with anti-CD3/anti-CD28 antibodies (ctrl) or cultured under Th17 polarizing conditions in the presence or absence of IL-33. **d** Frequencies of ST2-positive or **e** IL-17A-positive cells were measured among CD4^+^ FOXP3^+^ Tregs. **f** Flow cytometry plots from one representative experiment showing the gating strategy applied to assess the proportion of IL-17A-expressing cells among FOXP3^+^ (right upper panel) or FOXP3^-^ CD4^+^ T cells (right lower panel). In this particular dataset, eGFP^−^ CD4^+^ T were cultured under Th17-polarizing conditions, in the presence of IL-33. Frequencies of IL-17A-expressing cells were measured among **g** CD4^+^ FOXP3^+^ Tregs or FOXP3^−^ CD4^+^ T cells **h**. **d**, **e** Data are mean ± SEM and were pooled from two representative experiment (*n* = 6 mice). Statistical analyses were performed using paired Student’s *t*-test. **g**, **h** Data are mean ± SEM (*n* = 10 mice, pooled from three independent experiments) and statistical analyses were performed using Wilcoxon matched-pairs signed rank test. *P < 0.05; **P < 0.01

FOXP3^+^ Tregs exhibit phenotypic plasticity and provide a source of IL-17 in CRC.^27^ IL-17 contributes to intestinal tumorigenesis in mice since genetic ablation of *Il17a* leads to decreased AOM/DSS-induced CRC.^28^ Our transcriptomic analysis revealed that genes from the ontology pathway “Th17 cell differentiation” were differentially expressed in ST2^+^ versus ST2^−^ tumor-derived CD4^+^ FOXP3^+^ T cells (Fig. 4b and Supplementary Table 1). More precisely, *Il6ra*, *Rorc*, and *Il1r1* were downregulated in ST2^+^ CD4^+^ FOXP3^+^ Tregs (Fig. 5a), pointing towards an impaired ability to convert into IL-17-expressing cells. Congruent with these findings, IL-17 protein in CRC lesions was detected in ST2^−^ Tregs or CD4^+^ effector T cells, but not in ST2^+^ Tregs (Fig. 5b, c).

To further investigate whether IL-33 signaling may affect the ability of FOXP3^+^ Tregs to produce IL-17, we cultured CD4^+^ FOXP3^+^ T cells from naïve *Foxp3*/eGFP mice under Th17-polarizing conditions, with or without rmIL-33. Besides increasing ST2 expression, IL-33 inhibited the expression of IL-17A in CD4^+^ FOXP3^+^ Tregs (Fig. 5d, e). To assess whether IL-33 may also affect IL-17 expression of in vitro-induced Th17 cells, naïve CD4^+^ FOXP3^−^ T cells were in vitro differentiated into Th17 cells in the presence or absence of rmIL-33. Interestingly, Th17-polarizing conditions promoted the generation of IL-17A-producing CD4^+^ FOXP3^+^ Tregs, which was impaired upon addition of rmIL-33 (Fig. 5f, g). Furthermore, IL-33 signaling also reduced the proportion of IL-17A-expressing FOXP3^−^ (conventional) CD4^+^ T cells among Th17-polarized cells (Fig. 5h). These results are in agreement with our previous data indicating increased *Il17a* transcript levels in CRC lesions of *St2*^−*/−*^ versus WT mice (mean relative expression: 2.99 × 10^−2^ ± 9.25 × 10^−3^ versus 3.98 × 10^−4^ ± 1.44 × 10^−4^, *P* < 0.01; *n* = 9 and 8, respectively).^16^ Therefore, IL-33 appears to restrain the conversion of CD4^+^ T cells into IL-17-producing cells, also during intestinal tumorigenesis.

### ST2 deficiency promotes CD8^+^ T cell effector function and leads to improved tumor control in AOM/DSS-treated mice

**Fig. 6:**
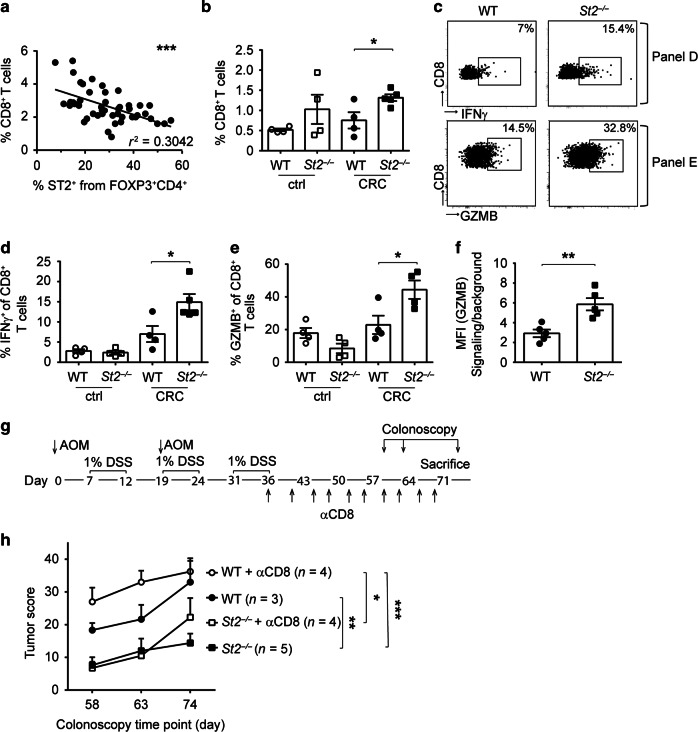
Increased frequencies of effector CD8^+^ T cells in CRC lesions of *St2*^−/−^ mice lead to improved antitumor immunity. Mice were treated with AOM/DSS and immune cells were analyzed in CRC lesions (CRC) or in adjacent tumor-free colons (ctrl). **a** Frequencies of CD8^+^ T cells were correlated with frequencies of ST2^+^ CD4^+^ Tregs in WT tumor tissues (*n* = 47 mice, pooled from nine independent experiments). **b** Frequencies of CD8^+^ T cells were measured in the intestine of the indicated strains. **c** Flow cytometry plots from one representative mouse per group depicted in **d** or **e** showing CRC-derived CD8^+^ T cells expressing IFNγ or GZMB, respectively. **d** Frequencies of IFNγ– or **e** GZMB-expressing CD8^+^ T cells were measured in the intestine of the indicated strains. **f** Median fluorescence intensity (MFI) of GZMB was measured on CRC-derived CD8^+^ T cells. **g** Mice were treated with AOM/DSS with or without antibody-mediated CD8^+^ T cell depletion, as shown in this experimental setup; **h** colonoscopy was then performed for longitudinal assessment of the tumor score in the indicated groups, at the indicated time points after the first AOM injection. Data are mean ± SEM. Data in **b–f** depict one representative out of six independent experiments (*n* = 4–5 mice per group) and data in **h** show one out of two experiments. Correlation in **a** was performed using Spearman correlation analysis and statistical analyses were performed using **b–f** standard Student’s *t-*test or **h** two-way ANOVA followed by uncorrected Fisher's LSD test. Statistics in **h** are shown only for the last colonoscopy time point. *P < 0.05; **P < 0.01; ***P < 0.001

Using the AOM/DSS-induced model of CRC, we previously demonstrated that cytotoxic CD8^+^ T cells critically participate in antitumor immunity, and that ablation of FOXP3^+^ Tregs during intestinal tumorigenesis results in an increase of effector CD8^+^ T cells in the colon.^19^ Remarkably, we found a negative correlation between the frequency of ST2^+^ CD4^+^ FOXP3^+^ T cells and the frequency of CD8^+^ T cells in AOM/DSS-induced CRC lesions, thereby suggesting that IL-33/ST2 signaling adversely affects CD8^+^ T cell immunity during CRC (Fig. 6a). Indeed, there was a higher proportion of CD8^+^ T cells in the colon of AOM/DSS-treated *St2*^*−/−*^ compared to WT mice (Fig. 6b). Furthermore, frequencies of IFNγ-expressing CD8^+^ T cells (Fig. 6c, d) and GZMB production in CD8^+^ T cells (Fig. 6c, e, f) were increased in *St2*^*−/−*^ versus WT CRC tissue.

Together, these results suggested that AOM/DSS-treated *St2*^*−/−*^ mice develop smaller CRC tumors compared to WT controls because of improved cytotoxic CD8^+^ T cell immunity in their colon. To address this possibility, we induced CRC in *St2*^−/−^ and WT mice and depleted CD8^+^ T cells by repetitively injecting anti-CD8 antibody after the last cycle of DSS (Fig. 6g). As expected, WT mice depleted of CD8^+^ T cells showed a tendency to higher tumor scores than CD8^+^ T cell-replete counterparts 63 days after treatment start (*P* = 0.0546). Importantly, long-term depletion of CD8^+^ T cells also triggered tumorigenesis in *St2*^*−/−*^ mice, to tumor scores comparable to the ones in non-depleted control WT mice (Fig. 6h).

Therefore, we conclude that IL-33/ST2 signaling on CD4^+^ FOXP3^+^ Tregs acts to restrain CD8^+^ T cell-mediated immune surveillance in the AOM/DSS model of CRC.

### Increased presence of ST2^+^ FOXP3^+^ Tregs in the blood and tumors of CRC patients

**Fig. 7:**
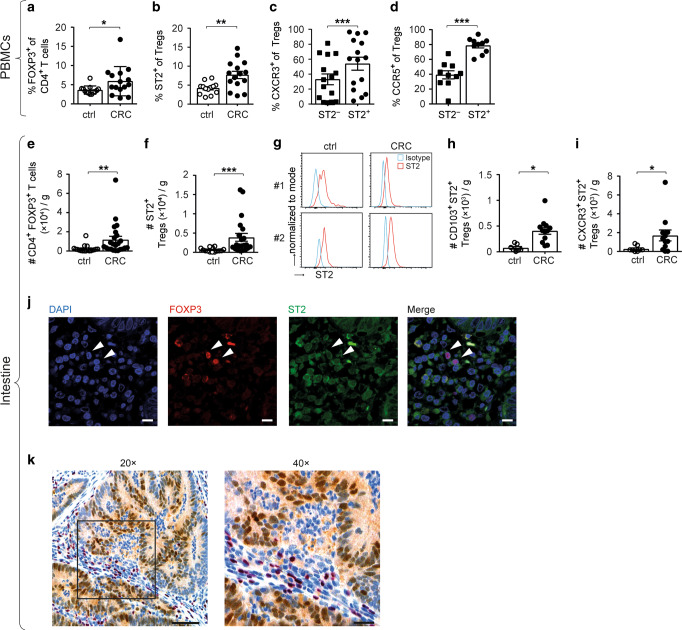
Increased presence of ST2^+^ FOXP3^+^ Tregs in the PBMCs and tumors of CRC patients. CD4^+^ FOXP3^+^ Tregs were analyzed in PBMCs from healthy controls (ctrl) and CRC patients to measure **a** cell frequencies and **b** proportions of ST2-expressing cells (ctrl, *n* = 13; CRC, *n* = 15). **c** Frequencies of CXCR3-expressing (*n* = 15) or **d** CCR5-expressing cells were quantified among circulating ST2-negative versus ST2-positive CD4^+^ FOXP3^+^ Tregs from CRC patients (*n* = 10). Alternatively, lymphocytes were isolated from tumor (CRC) versus adjacent tumor-free (ctrl) colons and **e** numbers of CD4^+^ FOXP3^+^ Tregs (ctrl, *n* = 18; CRC, *n* = 23) and of **f** ST2^+^ Tregs (ctrl, *n* = 14; CRC, *n* = 21) were determined per gram of tissue. **g** Representative histograms showing ST2 expression on CD4^+^ FOXP3^+^ Tregs from tumor versus adjacent tumor-free colons from two CRC patients. Numbers of intestinal CD4^+^ FOXP3^+^ Tregs were determined for **h** CD103^+^ (ctrl, *n* = 7; CRC, *n* = 12) or (**i**) CXCR3^+^ (ctrl, *n* = 8; CRC, *n* = 12) cells. **j** Immunofluorescence was performed on human CRC tissue for DAPI (blue), FOXP3 (red), and ST2 (green). Scale bars: 10 µm. **k** Representative immunohistochemistry of human CRC tissue showing FOXP3-expressing lymphocytes (red) in the vicinity of IL-33-positive transformed intestinal epithelial cells (brown). Image magnification is indicated. Scale bars: 100 and 50 µm, respectively. Data are mean ± SEM. Statistical analyses were performed using **a**, **b** Mann–Whitney test, **c**, **d** paired Student’s *t-*test, **e**, **f**, **h**, **i** Wilcoxon matched-pairs signed-rank test. *P < 0.05; **P < 0.01; ***P < 0.001

To address the general relevance of our findings from murine studies, we next assessed ST2 expression in FOXP3^+^ Tregs from blood and colon of CRC patients. Compared to healthy donors, we found increased proportions of CD4^+^ FOXP3^+^ T cells and ST2-positive Tregs in peripheral blood mononuclear cells (PBMCs) of CRC patients (Fig. 7a, b). In agreement with our mouse data, the migration markers CXCR3 and CCR5 were more frequently expressed on circulating ST2^+^ versus ST2^−^ CD4^+^ FOXP3^+^ Tregs from CRC subjects (Fig. 7c, d).

We next studied the immune cell infiltrate of resected colon cancers. Compared to adjacent tumor-free colon, CD4^+^ FOXP3^+^ T cells and ST2-positive Tregs were more abundant in human CRC lesions, yet ST2 expression levels were unchanged in tumor tissue (Fig. 7e–g). There were also more ITGAE (CD103)-expressing and CXCR3-expressing ST2^+^ Tregs in CRC than in adjacent tumor-free colon (Fig. 7h, i). Further studies using immunofluorescence confirmed the presence of ST2^+^ FOXP3^+^ cells in human intestinal tumors. Moreover, these FOXP3^+^ lymphocytes were located in the vicinity of IL-33-expressing tumor cells (Fig. 7j, k).

These findings extend our in vivo data in mice and substantiate their relevance for the human intestine. Moreover, they suggest that the IL-33/ST2 pathway acts on CD4^+^ FOXP3^+^ Tregs also in human CRC.

## Discussion

The tumor microenvironment has lately emerged as a major contributor to tumorigenesis, by providing either proliferative or inhibitory signals to the malignant cells.^29^ This is especially true for CRC, whose progression is substantially affected by cytokines and inflammatory mediators released in the tumor milieu.^30,31^ As an alarmin preferentially expressed in epithelial cells at barrier sites, and because of its role in intestinal disorders, IL-33 has recently gained particular attention for its contribution to CRC. However, while most reports describe a pro-tumorigenic effect of IL-33 in the intestine, several studies suggest the opposite (reviewed in refs. ^3,32^). Moreover, the functional role of IL-33 on ST2-expressing cells in the microenvironment of CRC has remained largely elusive.^32^

Here, we present evidence that IL-33/ST2 signaling shapes the phenotype of Tregs to promote intestinal tumorigenesis. Indeed, our data show that ST2-expressing Tregs preferentially accumulate in CRC lesions, and these cells are phenotypically distinct from ST2-negative counterparts. This leads to a restrained CD8^+^ T cell-mediated tumor immune surveillance, and thus an enhancement of intestinal tumorigenesis. In addition, we found that the IL-33/ST2 pathway suppresses IL-17 production and thereby modifies the inflammatory signals within the tumor microenvironment.

Tregs limit overt inflammatory responses to maintain intestinal immune homeostasis. However, the role of Tregs in CRC remains controversial. As a matter of fact, Treg suppressive activity may produce dual effects on inflammation-associated cancers, such as CRC, and tumor-infiltrating Tregs may correlate with either better or worse prognosis of patients with intestinal tumors (reviewed in ref. ^33^). In mice, Treg depletion during the early phase of the AOM/DSS treatment exacerbates the intestinal inflammation, leading to decreased survival. In contrast, Treg depletion at later stages curbs tumorigenesis.^19^

In human CRC lesions, ST2 protein is expressed not only on normal and transformed epithelial cells, but also on endothelial cells, myofibroblasts, and infiltrating immune cells.^15,16^ Indeed, our previous findings from studies with BM chimeric mice indicate that both radio-resistant and hematopoietic cells engage the IL-33/ST2 pathway to drive tumorigenesis in the colon, thereby decreasing the intestinal barrier and inducing protumorigenic cytokines.^16^ In murine adenomatous polyps, ST2-positive subepithelial myofibroblasts and mast cells react to IL-33 by secreting extracellular matrix components, proteases, and growth factors associated with intestinal tumor progression.^15^ Dissection of immune populations in AOM/DSS-treated mice revealed FOXP3^+^ Tregs to be the preponderant type of ST2-positive cells in CRC lesions. Moreover, we found that frequencies of ST2^+^ Tregs gradually increase during CRC development to correlate with tumor scores and IL-33 expression. Inversely, lower Treg proportions in intestinal tumors of *St2*^−/−^ mice are associated with reduced CRC development. Importantly, kinetic analysis indicated that ST2^+^ Tregs accumulate in tumors, particularly at later stages of AOM/DSS-dependent CRC. A possible involvement of ST2-expressing Tregs in human cancer has not been investigated so far. Here, we establish that circulating FOXP3^+^ Tregs from patients with intestinal cancer upregulate ST2, and that ST2^+^ FOXP3^+^ Tregs show preferential accumulation in tumor versus non-tumor colonic tissues. These findings further validate the pertinence of our observations from mouse studies. Together, our results suggest that ST2-expressing Tregs precipitate intestinal tumorigenesis.

Of note, another study recently reported an anti-tumoral effect of the IL-33/ST2 pathway on the development of sporadic CRC, with an increased frequency of tumor-infiltrating FOXP3^+^ Tregs in BM chimeric mice lacking ST2 expression on both the hematopoietic and the radio-resistant compartments.^34^ These differences with our investigation may rely on the type of CRC model applied—AOM only versus AOM and DSS treatment—or the genetic background of the *St2*^−/−^ mice used—BALB/c versus C57BL/6J. Nevertheless, our data indicate that ST2^+^ CD4^+^ FOXP3^+^ Tregs may also promote tumorigenesis in other murine models of CRC based on genetic ablation of tumor suppressor genes critically regulating intestinal cancer.

Importantly, our data indicate that CRC-derived Tregs display a distinct phenotype when expressing ST2. Indeed, compared to ST2^−^ counterparts, ST2^+^ Tregs in human and mouse CRC exhibit increased expression of integrins and several chemokine receptors. Some of these molecules like CXCR3, CCR5, CCR9, and GPR15 have been involved in gut homing, trafficking to, or retention in the intestine.^35–38^ CCR5, which we found to be more present on circulating ST2^+^ versus ST2^−^ Tregs in intestinal cancer patients, has been implicated in the recruitment of Tregs to solid murine tumors, including CRC.^39^ Moreover, there is evidence for a potential involvement of IL-33 in immune cell chemotaxis.^40^ Of note, ST2^+^ Tregs have been previously described to preferentially home outside of secondary lymphoid organs, especially to the lung, but also to the intestine, at steady-state.^41^ Taken together, these data strongly suggest that IL-33/ST2 signaling promotes the accumulation of Tregs to CRC lesions by modulating their migratory or tissue-retention properties.

Our findings from in vitro functional studies and from transcriptomic analyses of tumor tissues and CRC-isolated Tregs also indicate an implication of the IL-33/ST2 axis in the regulation of IL-17A expression by FOXP3^+^ Tregs and FOXP3^−^ CD4^+^ T cells. Indeed, our data support the notion that IL-33/ST2 signaling constrains IL-17A production while it favors the generation of IL-17-negative ST2^+^ FOXP3^+^ Tregs. Tregs represent a heterogeneous population comprising a minor subpopulation with developmental plasticity.^42^ As a matter of fact, human and murine Tregs in CRC lesions may also produce IL-17. These IL-17^+^ FOXP3^+^ Tregs express RORγt and exert immunosuppressive function, yet they display compromised anti-inflammatory properties and are pathogenic in the context of CRC.^27^ Interestingly, in mice challenged with ovarian cancer or CRC cells, Th17 (IL-17A^+^ FOXP3^−^) and IL-17A^+^ FOXP3^+^ cells can transdifferentiate into IL-17A-negative CD4^+^ FOXP3^+^ T cells with immunosuppressive function. Moreover, transcriptomic analysis and flow cytometry of IL-17A^+^ FOXP3^+^ cells indicated ST2 as one of the markers of this Th17-to-Treg cell conversion.^43^ Our findings of increased *Il17a* transcript levels in tumors of *St2*^*−/−*^ mice are also in line with the previous observation that absence of ST2-mediated signaling favors the expansion of IL-17-producing Th17 cells in the small intestine of mice treated with anti-CD3 antibody.^44^

Therefore, we propose that the IL-33/ST2 axis regulates T cell plasticity in CRC tissue, by stabilizing the phenotype of IL-17-negative FOXP3^+^ Tregs and potentially promoting the conversion of IL-17-producing CD4^+^ T cell types to these IL-17-negative (RORγt^−^) FOXP3^+^ Tregs. Yet, additional studies are warranted to definitively address the function of IL-33/ST2 signaling for CD4^+^ T cell plasticity in the intestinal tumor environment. Furthermore, given the dual, context-dependent effect of IL-17-producing CD4^+^ T cell types in CRC,^27,45^ it will be also relevant to further investigate the relative contribution of the IL-33 and IL-17 pathways to intestinal tumorigenesis.

Tregs impair tumor rejection by suppressing the cytotoxicity of tumor-specific CD8^+^ T cells.^46^ Consequently, Treg depletion leads to intratumoral accumulation of activated cytotoxic CD8^+^ T cells and tumor regression.^19^ In AOM/DSS-treated mice, these cytotoxic CD8^+^ T cells directly control CRC development.^19^ Although ST2 expression has been associated with an activated phenotype and an improved capacity of Tregs to restrain T cell proliferation in vitro,^41^ we did not detect difference in the suppressive function of ST2^+^ versus ST2^−^ Tregs in our model. This suggests that lower tumor Treg frequencies and numbers—rather than altered Treg suppression—underlie the higher proportion of activated cytotoxic CD8^+^ T cells found in CRC lesions of *St2*^*−/−*^ versus WT mice. Nevertheless, our data establish that CRC-derived ST2^+^ Tregs are transcriptionally distinct from their ST2^−^ counterparts, as they also show increased surface expression of PD-1, a molecule involved in Treg development, function, and stability.^47^

In conclusion, our findings indicate that IL-33/ST2 signaling shapes the cellular and the inflammatory composition of the tumor microenvironment to promote intestinal tumorigenesis. They also delineate the complexity of the interactions between the different immune cell types in the cancer stroma, and indicate that strategies for CRC treatment should best be designed to target multiple rather than single inflammatory pathways for therapeutic blockade.

## Methods

### Mice

All animal experiments at the University of Bern were performed in accordance with the Swiss Federal regulations and were approved by the Cantonal Veterinary Office. Mice strains used in Bern were all on C57BL/6 genetic background. All animal experiments in Essen were performed in accordance with the ethical principles and guidelines for scientific experiments and were approved by the local Landesamt für Natur-, Umwelt- und Verbraucherschutz (LANUV, North-Rhine-Westphalia, Germany). Mice strains used in Essen were either on a BALB/c or on a C57BL/6 genetic background.

*Il1rl1*^*tm1Anjm*^ (*Il1rl1*^−/−^ *or St2*^−/−^) mice were previously described^48^ and backcrossed on a C57BL/6J background. C57BL/6J and C57BL/6-*Foxp3*^*tm1Flv*^/J (termed *Foxp3*/RFP; JAX stock #008374) mice were purchased from Jackson Laboratories and subsequently bred in house.

*St2*^fl/fl^;*Foxp3-Cre* mice, with Treg-specific *St2* deletion, were generated by crossing *Il1rl1*^tm1c(KOMP)Wtsi^ (www.komp.org)^49^ with *Foxp3*^tm1(cre)Saka^^50^ mice. B6.129(Cg)-*Foxp3*^*tm3(DTR/GFP)Ayr*^/J (*Foxp3*^*DTR*^ mice) are commercially available and have been previously described.^51^ For chimera experiments, homozygous female *Foxp3*^*DTR*^ mice were used as BM donors. BALB/c mice were purchased from Envigo (Borchen, Germany). *Il1rl1*^*tm1Aki*^ (termed *St2*^−*/−*^) and C.Cg-*Foxp3*^*tm2Tch*^/J (termed *Foxp3*/eGFP)^52^ mice on a BALB/c background were bred and housed at the local animal facility of the University Hospital Essen.

For all experiments, non-randomized groups of 6–9-week-old animals were either co-housed (for females) or soiled bedding was exchanged weekly (for males), 2 weeks prior to and during experiments.

*Apc*^tm1Rak^ (*Apc*^+/1638N^) mice have been previoulsy described^53^ and were bred at the University of Bern. *Msh2*^tm2.1Rak^ × B6.SJL-Tg(Vil-cre)997Gum/J (*Msh2*^fl/fl^;*Villin-Cre*) animals,^54^ with conditional inactivation of *Msh2* in intestinal epithelial cells, were kindly provided by Dr. Kevin Haigis and Dr. Winfried Edelman and analyzed in Calgary. Experiments with *Msh2*^fl/fl^;*Villin-Cre* mice were performed in accordance with the ethical laws of Alberta/Canada and with protocols approved by the Health Sciences Animal Care Committee (protocol Number AC17-0090), following the guidelines from the Canadian Council for Animal Care.

### Generation of and experiments using BM chimeras

Recipient mice were irradiated twice with a dose of 650 cGy each after a recovery time of 4 h in between. Mice were then adoptively transfused with 2–5 × 10^6^ whole BM cells from donor mice, injected i.v., and antibiotic-treated with Bactrim (Roche) and Baytril (Bayer) in the drinking water for 2 weeks. Following antibiotic treatment, recipient mice were co-housed (for females) or soiled bedding was exchanged with untreated mice for 2 weeks prior to the AOM/DSS treatment to ensure a faster re-colonization of the intestinal flora of the BM recipient mice. AOM/DSS treatment of chimeras was initiated 6–8 weeks after BM reconstitution.

### Induction and assessment of intestinal tumors in mice

Intestinal tumors were induced by treating mice with AOM (Sigma, A5486) and DSS (MP Biomedicals, 0216011090), as previously described.^16,19^ Seven days after intraperitoneal AOM injection (10–12.5 mg/kg of body weight), mice were given 1–3% DSS in the drinking water for 5 days, followed by 7 days of water. Mice then received a second injection of AOM, which were followed by two cycles of 1–3% DSS and water. Starting 6 weeks after the first injection of AOM, colonoscopy was performed at different time points to evaluate tumor development in the distal part of the colon and to determine tumor scores. In brief, mice were anesthetized with isoflurane, a rigid miniature endoscope was inserted into the colon for visualization of the intestinal tissue. Tumor size and numbers were assessed from the digital video recording of the endoscopic procedure and tumor sizes were graded as follows; size grade 1: very small but detectable tumors; size grade 2: tumor covering up to one-eighth of the colonic circumference; size grade 3: tumor covering up to one-fourth of the colonic circumference; size grade 4: tumor covering up to half of the colonic circumference; and size grade 5: tumor covering more than half of the colonic circumference. Tumor scores were then calculated for each mouse by adding tumor numbers and the size grades of all tumors.

Tumor load for each mouse was determined by adding the volume of each tumor, which was measured with a pair of sliding calipers and calculated as (*W* × *L* × *H*)×*π*/6, as previously described.^16^ Mice were sacrificed for final analysis 10–12 weeks after the first AOM injection.

Tumors in *Msh2*^fl/fl^;*Villin-Cre* were weighed using a fine scale (Mettler Toledo).

### In vivo cell depletion

For the depletion of FOXP3^+^ Tregs from *Foxp3*^*DTR*^ immune cells, mice were injected i.p. with diphtheria toxin (DT; Sigma-Aldrich, 30 ng/g of body weight) twice a week, for 2.5 weeks.

For depletion of CD8^+^ T cells, mice were injected i.p. with 250 µg of anti-mouse CD8 antibody (Bio X cell, clone YTS 169.4), twice a week, for 5 weeks.

### Isolation of immune cells from murine BM and intestinal tissue

For the isolation of immune cells from BM, femurs were flushed with DMEM containing 10% FCS. Cells were then centrifuged and filtered through a 70 µm cell strainer (BD Biosciences, San Jose, CA).

For the isolation of immune cells from the murine intestine, colons were flushed with PBS to remove feces and then longitudinally sectioned. Murine colonic tissues were cut into 1 cm pieces and washed by rotation twice for 10 min at 37 °C with PBS containing 3 mmol/l EDTA. Tissues were then washed by rotation twice for 15 min at 37 °C with RPMI-1640 containing 1% FCS, 1 mmol/l EGTA and 1.5 mmol/l MgCl_2_. Tissues were subsequently rinsed with PBS, further minced and digested for 60 min at 37 °C in RPMI-1640 medium containing 20% FCS and 100 U/ml collagenase IV (Sigma-Aldrich). After digestion, single cells were separated from the remaining tissue using a 40 µm cell strainer and washed with RPMI-1640 before further use.

For the isolation of lymphocytes from freshly resected intestinal tumor or tumor-free tissues from CRC patients, the same protocol was used as for murine tissues. All human CRC tissues were provided by the Tissue Bank Bern.

### Flow cytometry

Blood-isolated or tissue-isolated leukocytes were stained using the marker-specific fluorochrome-labeled antibodies indicated in Supplementary Table 2. For intracellular detection of proteins, including FOXP3 (murine and human), IL-17A, Helios, Ki67, and GZMB, a FOXP3 Transcription Factor Staining Buffer Kit was used (ThermoFisher). For the assessment of intracellular IL-17A or IFNγ, cells were stimulated with 1 µg/ml ionomycin and 10 ng/ml phorbol 12-myristate 13-acetate (PMA) in the presence of 5 µg/ml Brefeldin A (all Sigma-Aldrich). After staining with anti-CD8 antibodies, the BD Cytofix/Cytoperm Kit (BD Biosciences) was used for cell fixation and permabilization.

Samples were acquired using LSRII SORP, FACSCanto, or FACSCelesta instruments (all BD Biosciences) and analyzed using a DIVA (all BD Biosciences) or FlowJo (version 7.2.5, Tree Star) software.

### T cell inhibition assay

For T cell inhibition assays, CD4^+^ FOXP3^+^ (eGFP^+^) Tregs were isolated from CRC lesions from *Foxp3*/eGFP mice and divided into ST2^+^ or ST2^−^ cell populations using a FACSAria II cell sorter (BD Biosciences). As responder cells, CD4^+^ T cells were MACS-purified from spleens of naive *St2*^*−/−*^ mice (BALB/c background) using a CD4^+^ T Cell Isolation Kit II (Miltenyi Biotec) and labeled with eFluor670 (Thermo Fisher) following the manufacturer’s instructions. eFluor670-labeled responder CD4^+^ T cells (5 × 10^4^) were either cultured alone or co-cultured with CD4^+^ FOXP3^+^ (eGFP^+^) ST2^+^ or ST2^−^ Tregs (2.5 × 10^4^) for 3 days in the presence of 1 μg/ml anti-CD3 (clone 145-2C11; BD Biosciences). Irradiated splenocytes from naïve *St2*^−*/−*^ mice served as antigen-presenting cells (1.5 × 10^5^). Where indicated, 30 ng/ml rmIL-33 (BioLegend) was added to the culture.

### In vitro Th17 cell differentiation

CD4^+^ T cells were isolated from spleens of *Foxp3*/eGFP mice using the CD4^+^ T Cell Isolation Kit II (Miltenyi Biotec) according to the manufacturer’s protocol. CD4^+^ T cells were divided into eGFP^+^ or eGFP^−^ cell populations using a FACSAria II cell sorter (BD Biosciences). As a control, eGFP^−^ CD4^+^ T cells (5 × 10^5^) or eGFP^+^ CD4^+^ Tregs (1 × 10^5^) were stimulated with 1 μg/ml anti-CD3 (clone 145–2C11; BD Biosciences) and 1 μg/ml anti-CD28 (clone 37.51; BD Biosciences). To induce Th17 polarization, cells were in addition incubated with 50 ng/ml recombinant murine (rm) IL-6, 20 ng/ml rmIL-1β, 100 ng/ml rmIL-21, 20 ng/ml rmIL-23, 2 ng/ml recombinant human TGF-β, 200 ng/ml anti-IL-4, 200 ng/ml anti-IL-2, and 200 ng/ml anti-IFNγ. Where indicated, cells were further cultured with 10 ng/ml recombinant murine IL-33 (rmIL-33; BioLegend). Fresh media supplemented with Th17-polarizing cytokines and rmIL-33 was added on day 3, and cells were analyzed for IL-17A expression on day 4 (eGFP^+^ CD4^+^ Tregs) or on day 5 (eGFP^−^ CD4^+^ T cells).

### Quantitative PCR assay

Mouse colonic tissue was homogenized using a TissueLyser device (Qiagen). RNA was extracted using TRI-reagent (Sigma-Aldrich) or an RNeasy Fibrous Tissue Kit (Qiagen). RNA was then reverse-transcribed into cDNA using an M-MLV Reverse Transcriptase (Promega) and a mixture of oligo(dT) and random hexamer primers. For the qPCR reaction, FastStart SYBR Green Master (Roche Diagnostics) or a Maxima SYBR Green qPCR Master Mix (Fermentas/Thermo Fisher) were used with commercial primers specific for *St2* or *Il33* (Qiagen). Alternatively, self-designed primers were used to detect *sSt2* (Fwd: 5′-TCG AAA TGA AAG TTC CAG CA-3′; Rev: 5′-TGT GTG AGG GAC ACT CCT TAC-3′) or *Il33* (Fwd: 5′-CTA CTG CAT GAG ACT CCG TTC TG-3′; Rev: 5′-AGA ATC CCG TGG ATA GGC AGA G-3′). Reactions were run on a StepOnePlus Real Time PCR System (Life Technologies/Thermo Fisher) or on an ABI PRISM Sequence Detection System (Applied Biosystems/Thermo Fisher). Transcript expression levels were normalized to *Gapdh* mRNA, and control versus CRC tissue were compared applying the 2^–ΔΔCT^ method. Alternatively, expression levels of target genes were determined with included standard curves for each individual gene and further normalization to the housekeeping gene *Rps9*.

### Cytokine measurement in serum and tissues

Protein levels in serum samples from CRC patients or controls were measured using a commercial ELISA kit for sST2 (R&D Systems) or by flow cytometry using a bead-based multiplex assay (LEGENDplex^TM^ human inflammation 13-plex panel; BioLegend).

IL-33 in the supernatants of murine colonic explant cultures was measured by polystyrene bead-based Luminex technology (R&D Systems) according to the manufacturer’s instructions. A Luminex 200 instrument (Luminex Corporation) was used to run the assay.

### Gene expression and pathway analysis

For gene expression analysis, CD4^+^ FOXP3^+^ (eGFP^+^) Tregs were isolated from CRC lesions form *Foxp3*/eGFP mice and divided into ST2^+^ or ST2^−^ cell populations using a FACSAria II cell sorter (BD Biosciences). A total of 0.5–1.5 × 10^5^ ST2^+^ or ST2^−^ Tregs were homogenized in RLT buffer (Qiagen) and RNA was isolated with the RNeasy kit (Qiagen). The quality and integrity of total RNA were controlled with an Agilent 2100 Bioanalyzer (Agilent Technologies). Next, 2–10 ng of total RNA were used for biotin labeling with the GeneChip Pico Kit (Affymetrix). Samples were hybridized to Clariom™ S chips (Affymetrix) and stained in the Affymetrix Fluidics Station 450 according to the manufacturer’s recommendations. Microarrays were scanned with an Affymetrix GCS 3000 scanner running with Affymetrix GeneChip Command Console Software (AGCC) and Affymetrix Expression Console™ Software.

RNA microarray data from tumor-isolated ST2^+^ versus ST2^−^ CD4^+^ FOXP3^+^ (eGFP^+^) Tregs can be downloaded from the ArrayExpress Archive of Functional Genomics Data (https://www.ebi.ac.uk/arrayexpress/experiments/E-MTAB-6842/; name: “Pairwise comparison of ST2+ and ST2- FOXP3+ T cells derived from mouse intestinal tumors”)

For the analysis of differentially expressed pathways, a gene set enrichment analysis (GSEA) was performed using the SetRank method.^55^ This method first calculates the *p*‐value of a gene set utilizing the ranking of its genes in the ordered list of *p*‐value as determined by limma. Next, it discards gene sets that have been initially flagged as significant, if their significance is merely due to the overlap with another gene set. Gene sets were derived from the following databases: BIOCYC, Gene Ontology, ITFP, KEGG, LIPID MAPS, PhosphoSitePlus, REACTOME, and WikiPathways. Supplementary Table 3 shows the data output from the limma analysis and Supplementary Table 4 provides a list of all pathways detected by the SetRank method.

### Isolation of peripheral blood mononuclear cells from human donors

Blood samples from CRC patients or healthy donors were collected in NH_4_-Heparin Monovette tubes (Sarstedt). PBMCs were isolated by Ficoll density gradient (Biochrom AG) centrifugation. Isolated cells were washed with PBS containing 2 mmol/l EDTA and 2% FCS (PAA Laboratories), and cryopreserved in cell culture medium containing 10% FCS and 10% DMSO (Carl Roth GmbH) until further analysis.

Patients and healthy donors gave written informed agreement and analyses of the samples were approved by the Cantonal Ethics Committee of Bern (2017-01821) or the Ethics Committee of the Medical Faculty of the University Duisburg Essen (AZ 05-2882).

### Histology and immunohistochemistry

Human tissue sections were fixed in 4% formaldehyde and embedded in paraffin. All staining reactions were performed by automated staining using a BOND RX autostainer (Leica Biosystems).

For double immunohistochemistry, sections were deparaffinized and antigen was retrieved using 1 mM Tris solution (pH 9.0) for 30 min at 95 °C. Sections were stained with goat anti-human IL-33 (R&D Systems) and mouse anti-human FOXP3 (eBioscience) primary antibodies, at a dilution of 1:400 and 1:100, respectively. As secondary antibodies, a rabbit anti-goat (Dako) antibody were used at a dilution of 1:400. Specific binding of primary antibodies was visualized using a polymer-based visualizing system with horseradish peroxidase as the enzyme and 3,3-diaminobenzidine (DAB) as a brown chromogen, or an alkaline phosphatase-linked polymer and Fast Red as red chromogen (Bond™ Polymer Refine Red Detection), respectively (all from Leica Biosystems). Finally, the samples were counterstained with haematoxylin and mounted with Aquatex (Merck). Slides were scanned using a Pannoramic 250 digital scanner (3DHISTECH).

For double immunofluorescence staining, antigen retrieval was performed using a citrate buffer-based solution (pH 6.0) for 30 min at 100 °C. Rabbit anti-human ST2 (ABclonal) and mouse anti-human FOXP3 (eBioscience) were applied as primary antibodies at a dilution of 1:500 or 1:50, respectively. As secondary antibodies, an Alexa Fluor 488-conjugated goat anti-rabbit (Cell Signaling Technology) and an Alexa Fluor 555-conjugated goat anti-mouse (Thermo Fisher) antibody were used, both at a dilution of 1:1000. Digital pictures were taken using a LSM 700 laser scanning confocal microscope and an AxioCam MRm digital camera (both from Zeiss).

### Statistical analysis

Results were tested for normal distribution using D'Agostino and Pearson omnibus normality test. For single comparison of normally distributed data, two-tailed standard or paired Student’s *t*-test was used. For single comparison of not normally distributed data, Mann–Whitney or Wilcoxon matched-pairs signed rank test was used. When means of more than two groups were compared, one-way ANOVA with Dunnett's post-test or Bonferroni post-test, or two-way ANOVA with Sidak post-test or uncorrected Fisher's LSD test were used. Statistical analyses were performed using GraphPad Prism v. 6.03 or v. 7.03 for Windows (Graph-Pad Software). Unless specified, only statistically significant differences are indicated in the figures. For all statistical analyses: * *P* < 0.05; **P < 0.01; ***P < 0.001;****P < 0.0001.

## Supplementary Information

Supplementary Information

Supplementary Data 1

Supplementary Data 2
