# Supplementary material for: The IL-33/ST2 pathway shapes the regulatory T cell phenotype to promote intestinal cancer
Source: Mucosal Immunol. 2019 Jun 5;12(4):990–1003. doi: 10.1038/s41385-019-0176-y (PMC7746527; doi:10.1038/s41385-019-0176-y)
Supplement: Supplementary file 1 — Supplementary Information [file 41385_2019_176_MOESM1_ESM.pdf]

Supplementary Information

Supplementary Figure 1

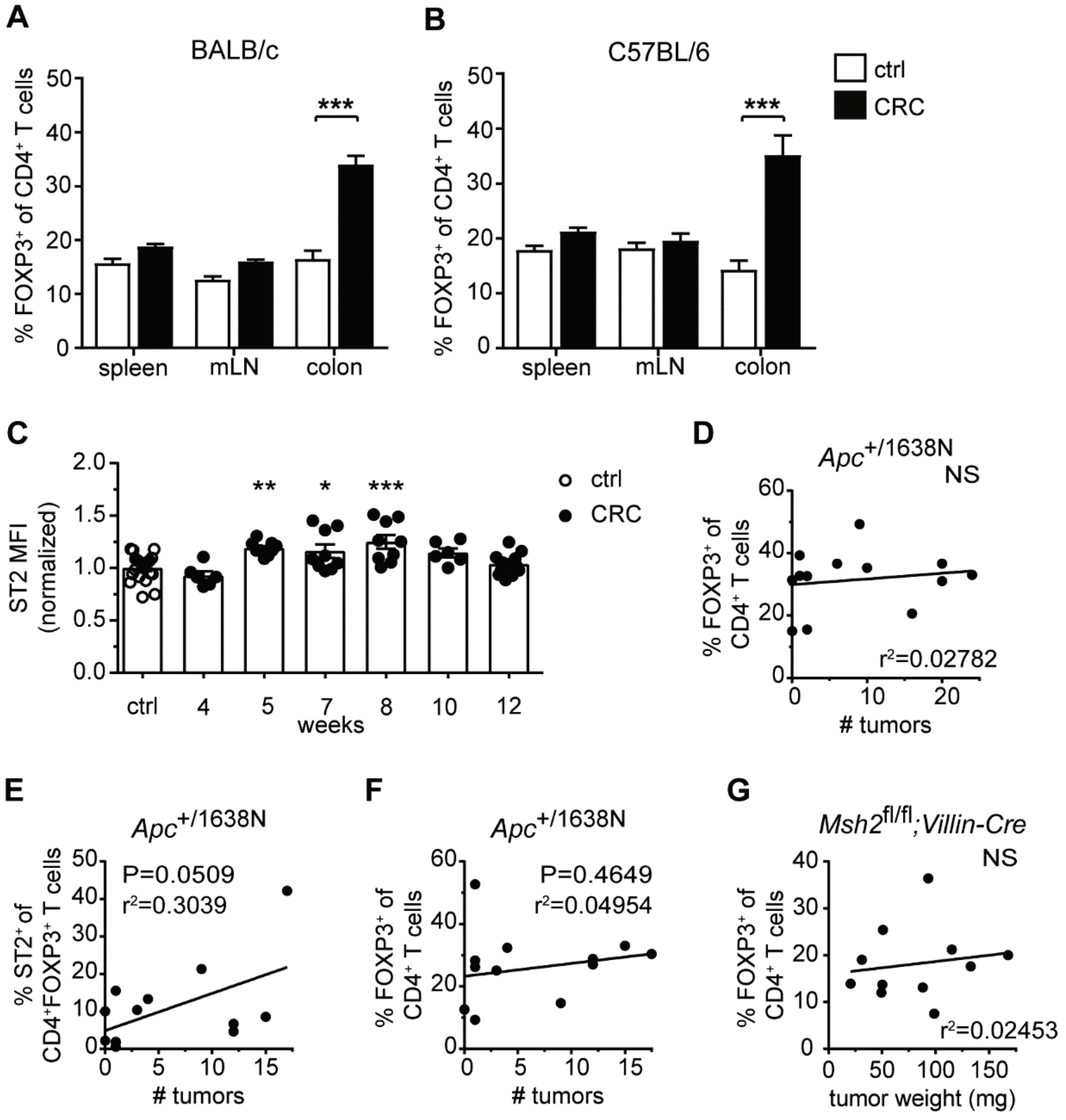

$FOXP3^+$ Tregs preferentially accumulate in AOM/DSS-induced CRC lesions but do not correlate with tumorigenesis in murine genetic models of CRC.

(A) BALB/c (n=7-8 mice per group) or **(B)** C57BL/6 (n=15-22 animals per group) mice were treated with AOM/DSS (CRC) or left untreated (ctrl), and frequencies of $CD4^+$ $FOXP3^+$ Tregs cells were analyzed in spleen, mesenteric lymph nodes (mLN) or colon. **(C)** ST2 mean fluorescence intensity (MFI) relative to isotype control was measured on $ST2^+$ $FOXP3^+$ Tregs isolated from CRC lesions of BALB/c mice at the indicated time points during AOM/DSS treatment (n=20 for ctrl and n=6-12 for CRC mice per time point). Alternatively, *Apc*$^{+/1638N}$

mice (n=13) were analyzed at different ages and frequencies of $ST2^+$ $FOXP3^+$ or $FOXP3^+$ Treg were correlated with tumor numbers in the **(D)** colon or **(E, F)** small intestine. **(G)** *Msh2*$^{fl/fl}$;*Villin-Cre* mice (n=11) were analyzed 269-342 days after birth and frequencies of $FOXP3^+$ Treg were correlated with tumor weight in the small intestine. Data are mean ± SEM and were pooled from **(A, G)** two, **(B)** four, **(C)** three or **(D-F)** several independent experiments. Statistical analyses were performed using **(A, B)** two-way ANOVA with Sidak post-test or **(C)** one-way ANOVA with Dunnett's post-test, and correlations were calculated using **(D, G)** Spearman or **(E, F)** Pearson correlation analysis. NS, non-significant. *: $P<0.05$; **: $P<0.01$; ***: $P<0.001$.

Supplementary Figure 2

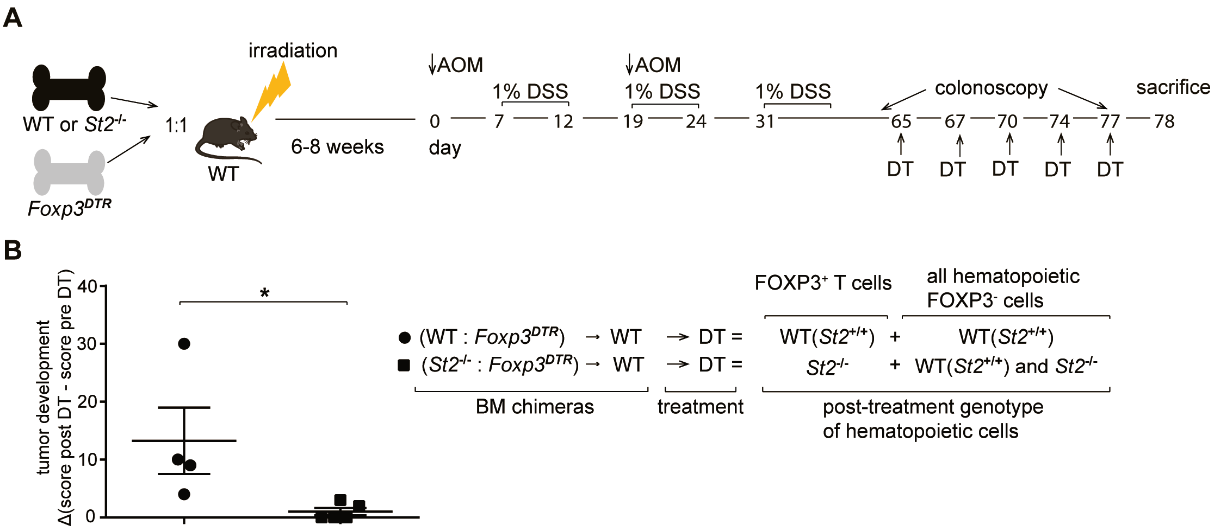

FOXP3⁺ Tregs-restricted St2 deficiency leads to improved CRC control.

Indicated sets of mixed chimeric mice were treated with AOM/DSS and injected i.p. with diphtheria toxin (DT) to deplete DT receptor-expressing FOXP3^{+} Tregs. **(A)** Experimental setup. **(B)** Colonoscopy was performed to assess tumor score at different time points, and tumor development was calculated by subtracting tumor score before from tumor score post DT application. Data are mean ± SEM (n=4-5 mice per group). Statistical analysis was performed using Mann-Whitney test. *: P<0.05.

Supplementary Figure 3

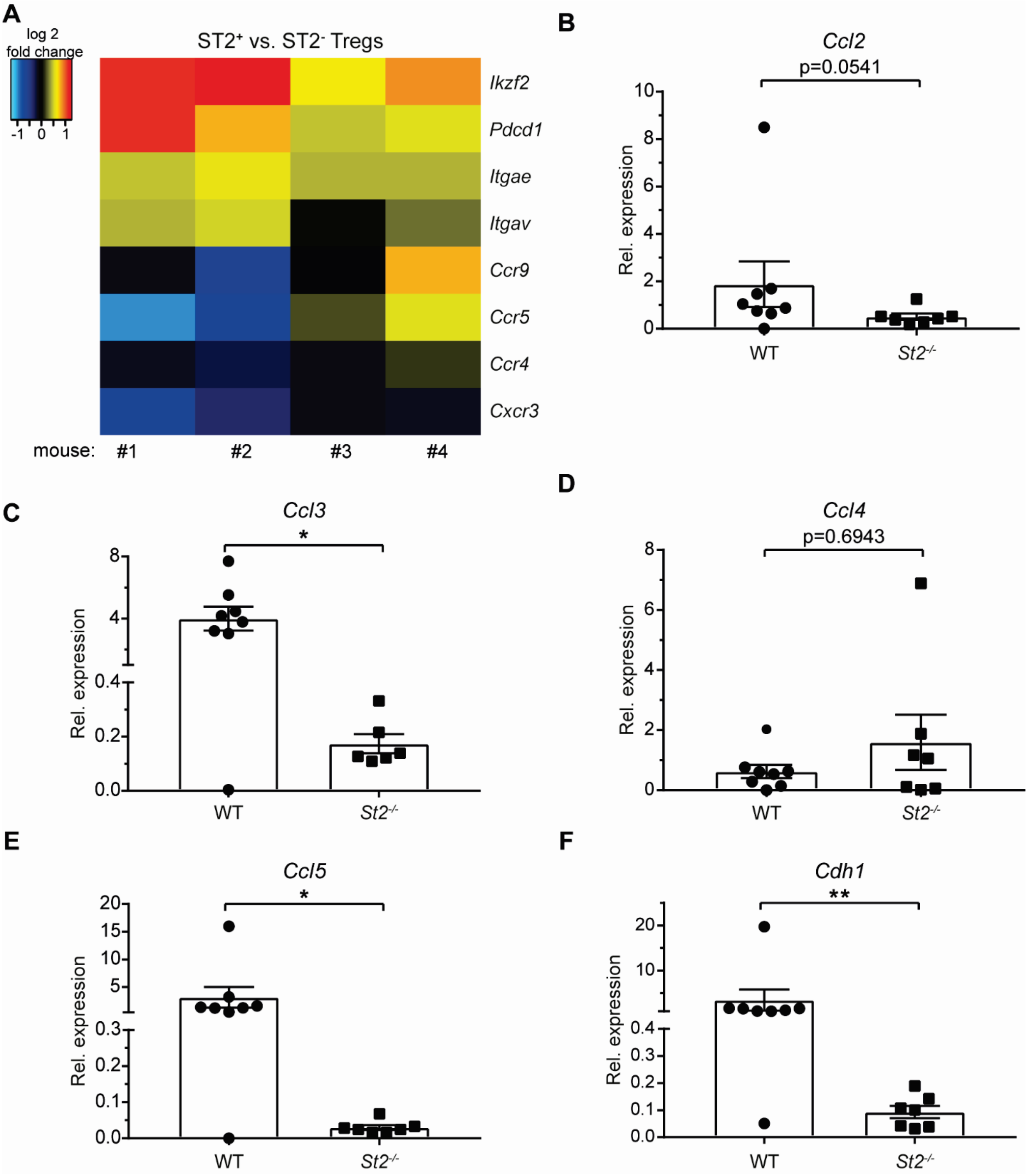

ST2 modulates the expression of migration-associated molecules in tumor-derived FOXP3⁺ Tregs and in CRC lesions.

(A) Heat map showing expression levels of selected genes in the gene ontology pathway "leukocyte migration" (GO:0050900) in ST2^{+} versus ST2^{-} eGFP^{+} Tregs isolated from the intestine of the indicated four mice, after AOM/DSS treatment. Blue indicates higher transcript

expression in ST2$^-$ Tregs and red higher expression in ST2$^+$ Tregs. *Ikzf2*, *Pdcd1*, and *Itgae* are differentially expressed in tumor-derived ST2$^+$ versus ST2$^-$ Tregs (adjusted p value p≤0.05). Alternatively, WT versus *St2*$^{-/-}$ mice were treated with AOM/DSS and transcript levels were quantified in CRC lesions of WT versus *St2*$^{-/-}$ mice for **(B)** *Ccl2*, **(C)** *Ccl3*, **(D)** *Ccl4*, **(E)** *Ccl5* and **(F)** *Cdh1*. For panels **(B-F),** data represent means ± SEM and statistical analyses were performed using Mann-Whitney test (n=6-8 mice per group). *: P<0.05, **: P<0.01.

Supplementary Table 1

Table listing the 58 gene ontology pathways that are differentially expressed in $ST2^+$ versus $ST2^-$ $eGFP^+$ Tregs isolated from CRC lesions (with adjusted p value p<0.001 and corrected p value p<0.001). Related to the data depicted in Figure 4.

name	description	trivial name	database	size	pSetRank	correctedPValue	adjustedPValue
GO:0032496	response to lipopolysaccharide	LPS resp.	GOBP	177	1,93E-025	3,50E-010	1,26E-008
GO:1903706	regulation of hemopoiesis	Hemo. reg.	GOBP	249	4,38E-015	1,04E-004	1,26E-008
ko04659	Th17 cell differentiation	Th17 dif.	KEGG	88	2,77E-012	2,26E-005	1,26E-008
GO:0050900	leukocyte migration	Leuk. migration	GOBP	145	1,92E-011	9,34E-007	1,26E-008
GO:0006412	translation	transl.	GOBP	187	1,19E-008	5,76E-009	1,26E-008
GO:0030098	lymphocyte differentiation	Lymph. dif.	GOBP	207	1,30E-008	2,29E-006	1,26E-008
R-MMU-198933	Immunoregulatory interactions between a Lymphoid and a non-Lymphoid cell	Lymph. Inter.	REACTOME	77	4,05E-007	2,43E-009	1,26E-008
ko00190	Oxidative phosphorylation	OXPHOS	KEGG	92	7,37E-005	1,04E-007	1,26E-008
WP373	IL-3 Signaling Pathway	IL-3 sign.	WikiPathways	80	1,88E-004	3,71E-005	1,26E-008
GO:0050727	regulation of inflammatory response	Inflam. resp. reg.	GOBP	216	5,30E-004	2,56E-004	1,26E-008
GO:0022409	positive regulation of cell-cell adhesion	Cell-cell adh.	GOBP	175	3,89E-003	9,29E-004	1,26E-008
R-MMU-6791226	Major pathway of rRNA processing in the nucleolus and cytosol	rRNA proces.	REACTOME	56	1,20E-002	1,26E-007	1,26E-008
R-MMU-1433557	Signaling by SCF-KIT	SCF-KIT sign.	REACTOME	230	6,69E-002	1,99E-004	1,26E-008
M00147	NADH dehydrogenase (ubiquinone) 1 beta subcomplex	NADH deh. 1β	KEGG	12	1,00E+000	5,24E-009	1,26E-008
GO:0051082	unfolded protein binding	Unfold. prot. bind.	GOMF	62	1,00E+000	1,98E-007	1,26E-008
R-MMU-69206	G1/S Transition	G1/S Transition	REACTOME	81	1,00E+000	9,19E-007	1,26E-008
M00355	Spliceosome, 35S U5-snRNP	Splice. U5-snRNP	KEGG	24	1,00E+000	2,53E-006	1,26E-008

R-MMU-975576	N-glycan antennae elongation in the medial/trans-Golgi	N-gly. elong.	REACTOME	19	1,00E+000	7,64E-006	1,26E-008
GO:0072678	T cell migration	Tc migration	GOBP	13	1,00E+000	4,40E-005	1,26E-008
GO:0045862	positive regulation of proteolysis	Proteolysis reg.	GOBP	214	1,00E+000	5,02E-005	1,26E-008
GO:0031072	heat shock protein binding	HSP bind.	GOMF	86	1,00E+000	7,20E-005	1,26E-008
GO:0002761	regulation of myeloid leukocyte differentiation	Myeloid leuk. dif.	GOBP	94	1,00E+000	9,22E-005	1,26E-008
GO:1903205	regulation of hydrogen peroxide-induced cell death	H2O2 cel. death	GOBP	27	1,00E+000	9,99E-005	1,26E-008
WP387	IL-6 signaling Pathway	IL-6 sign.	WikiPathways	78	1,00E+000	1,01E-004	1,26E-008
GO:0036438	maintenance of lens transparency	Lens transp.	GOBP	3	1,00E+000	1,07E-004	1,26E-008
R-MMU-5668541	TNFR2 non-canonical NF-kB pathway	TNFR2 path.	REACTOME	76	1,00E+000	1,47E-004	1,26E-008
R-MMU-114508	Effects of PIP2 hydrolysis	PIP2 hydrol.	REACTOME	21	1,00E+000	1,69E-004	1,26E-008
GO:0043122	regulation of I-kappaB kinase/NF-kappaB signaling	NFκB sign.	GOBP	166	1,00E+000	1,90E-004	1,26E-008
GO:0042254	ribosome biogenesis	Ribosome bio.	GOBP	159	1,00E+000	2,11E-004	1,26E-008
GO:0043161	proteasome-mediated ubiquitin-dependent protein catabolic process	Ub. catab.	GOBP	165	1,00E+000	3,02E-004	1,26E-008
GO:0042273	ribosomal large subunit biogenesis	Large ribosome bio.	GOBP	34	1,00E+000	3,20E-004	1,26E-008
GO:0032956	regulation of actin cytoskeleton organization	Cytosk. org.	GOBP	212	1,00E+000	3,47E-004	1,26E-008
ko04060	Cytokine-cytokine receptor interaction	Cytokine/rec.	KEGG	200	1,00E+000	3,57E-004	1,26E-008
ko04071	Sphingolipid signaling pathway	Sph.lipid sign.	KEGG	101	1,00E+000	3,97E-004	1,26E-008
GO:0017124	SH3 domain binding	SH3 bind.	GOMF	90	1,00E+000	4,42E-004	1,26E-008
GO:0030139	endocytic vesicle	Endo. ves.	GOCC	100	1,00E+000	4,90E-004	1,26E-008
ko03040	Spliceosome	Splice.	KEGG	87	1,00E+000	5,04E-004	1,26E-008
GO:0043021	ribonucleoprotein complex binding	RNP bind.	GOMF	91	1,00E+000	5,07E-004	1,26E-008
GO:0030667	secretory granule membrane	Sec. granule	GOCC	59	1,00E+000	5,31E-004	1,26E-008
GO:0090197	positive regulation of chemokine secretion	Chemokine sec.	GOBP	10	1,00E+000	5,32E-004	1,26E-008
GO:0002762	negative regulation of myeloid leukocyte differentiation	Neg. myeloid leuk. dif.	GOBP	37	1,00E+000	5,36E-004	1,26E-008
R-MMU-72689	Formation of a pool of free 40S subunits	40S sub.	REACTOME	22	1,00E+000	5,42E-004	1,26E-008
M00146	NADH dehydrogenase (ubiquinone) 1 alpha subcomplex	NADH deh. 1α	KEGG	14	1,00E+000	5,55E-004	1,26E-008

R-MMU-72695	Formation of the ternary complex, and subsequently, the 43S complex	43S complex	REACTOME	14	1,00E+000	5,73E-004	1,26E-008
M00337	Immunoproteasome	Immunoprot.	KEGG	14	1,00E+000	6,06E-004	1,26E-008
M00340	Proteasome, 20S core particle	20S proteasome	KEGG	10	1,00E+000	6,06E-004	1,26E-008
WP1496	Oxidative Damage	Ox. dam.	WikiPathways	29	1,00E+000	6,46E-004	1,26E-008
R-MMU-1169091	Activation of NF-kappaB in B cells	NFκB in Bc	REACTOME	49	1,00E+000	6,87E-004	1,26E-008
mmu04068	FoxO signaling pathway	FoxO sign.	KEGG	102	1,00E+000	6,93E-004	1,26E-008
GO:0002113	interleukin-33 binding	IL-33 binding	GOMF	1	1,00E+000	7,02E-004	1,26E-008
GO:0001503	ossification	ossif.	GOBP	171	1,00E+000	7,52E-004	1,26E-008
GO:0006606	protein import into nucleus	Nucl. Import	GOBP	86	1,00E+000	7,90E-004	1,26E-008
ko04210	Apoptosis	Apopt.	KEGG	107	1,00E+000	8,07E-004	1,26E-008
R-MMU-446203	Asparagine N-linked glycosylation	Asp. N-gly.	REACTOME	175	1,00E+000	8,77E-004	1,26E-008
GO:0051250	negative regulation of lymphocyte activation	Neg. reg. lymph.	GOBP	104	1,00E+000	9,00E-004	1,26E-008
GO:0030108	HLA-A specific activating MHC class I receptor activity	MHC I rec. activity	GOMF	1	1,00E+000	9,57E-004	1,26E-008
GO:0032154	cleavage furrow	cleav. furrow	GOCC	25	1,00E+000	9,70E-004	1,26E-008
GO:0007157	heterophilic cell-cell adhesion via plasma membrane cell adhesion molecules	Cell-cell adh.	GOBP	41	1,00E+000	9,98E-004	1,26E-008

Supplementary Table 2 (Antibodies, clones, conjugates and manufacturers)

Murine

Specificity	Clone name	Conjugated to	Catalog number	Source
CD4	RM4-4	Pacific Blue	116008	Biolegend
CD4	RM4-5	Pacific Blue	558107	BD Biosciences
CD4	RM4-5	BrilliantViolet 650	563747	BD Biosciences
CD4	GK15	APC	100412	BioLegend
CD8α	53-6.7	AlexaFluor700	45-5981-80	BioLegend
CD8α	53-6.7	BrilliantViolet 510	100752	BioLegend
CD8α	53-6.7	FITC	100706	BioLegend
CD11c	HL3	V450	560521	BD Biosciences
CD335	29.A1.4	Per-CP/Cy5.5	137610	BioLegend
CD335	29.A1.4	V450	560763	BD Biosciences
B220	RA3-6B2	PE	561878	BD Biosciences
F4/80	BM8	PE	12-4801-82	ThermoFisher
FOXP3	FJK-16s	FITC	11-5773-82	ThermoFisher
CD62L	MEL-14	APC-Cy7	104428	BioLegend
CD25	PC61	Per-CP/Cy5.5	102030	BioLegend
ST2	DIH9	APC	145305	BioLegend
ST2	DIH9	PerCP-Cy5.5	145311	BioLegend
ST2	RMST2-2	APC	17-9335-82	ThermoFisher
ST2	RMST2-2	PE-Cy7	25-9335-82	ThermoFisher
Fixable viability dye (FVD)		eFluor506	65-0866-14	ThermoFisher
Fixable viability dye (FVD)		eFluor780	65-0865-14	ThermoFisher
GZMB	GB11	Pacific Blue	515408	BioLegend
CD103	2E7	PerCP-Cy5.5	121415	BioLegend
CD103	M290	PE	557495	BD Biosciences
PD-1	J43	PE-Cy7	25-9985	ThermoFisher

PD-1	RMP1-30	PE-Cy7	109110	BioLegend
IFNγ	XMG1.2	APC	505810	BioLegend
IL-17A	TC11-18H10	PE	559502	BD Biosciences
CD51	RMV-7	PE	104105	BioLegend
Helios	22F6	eFluor450	48-9883-42	ThermoFisher
CXCR3	CXCR3-173	PE	12-1831-82	ThermoFisher
CCR4	2G12	PE-Cy7	131213	BioLegend
CCR5	HM-CCR5	PE	12-1951-81	ThermoFisher
CCR9	9B1	AlexaFluor647	129709	BioLegend
Ki67	SolA15	PE-Cy7	25-5698-80	ThermoFisher

Human

Specificity	Clone name	Conjugated to	Catalog number	Source
ST2	B4E6	FITC	101002F	MD Bioproducts
CD4	RPA-T4	PE	12-0049-41	ThermoFisher
CD4	RPA-T4	Pacific Blue	558116	BD Biosciences
FOXP3	PCH101	APC	17-4776-41	ThermoFisher
FOXP3	PCH101	PE	12-4776-42	ThermoFisher
CXCR3	CEW33D	PE-Cy7	25-1839-42	ThermoFisher
CXCR3	CEW33D	PE	12-1839-42	ThermoFisher
CD103	Ber-ACT8	BrilliantViolet 605	350217	BioLegend
CCR5	HEK/1/85a	Alexa Fluor® 647	313712	BioLegend

Supplementary Table 3 (microarray – gene expression)

Data output from the limma analysis for gene expression data in ST2+ versus ST2- Tregs from intestinal tumors (see attached .xls file).

<u>Supplementary Table 4 (microarray – pathway analysis)</u>

List of all pathways detected by the SetRank method upon analysis of the microarray data from ST2+ versus ST2- Tregs from intestinal tumors (see attached .xls file).
